# INDUS - a composition-based approach for rapid and accurate taxonomic classification of metagenomic sequences

**DOI:** 10.1186/1471-2164-12-S3-S4

**Published:** 2011-11-30

**Authors:** Monzoorul Haque Mohammed, Tarini Shankar Ghosh, Rachamalla Maheedhar Reddy, Chennareddy Venkata Siva Kumar Reddy, Nitin Kumar Singh, Sharmila S Mande

**Affiliations:** 1 Bio-sciences R&D Division, TCS Innovation Labs, Tata Consultancy Services Limited, 1 Software Units Layout, Madhapur, Hyderabad – 500081, Andhra Pradesh, India

## Abstract

**Background:**

Taxonomic classification of metagenomic sequences is the first step in metagenomic analysis. Existing taxonomic classification approaches are of two types, similarity-based and composition-based. Similarity-based approaches, though accurate and specific, are extremely slow. Since, metagenomic projects generate millions of sequences, adopting similarity-based approaches becomes virtually infeasible for research groups having modest computational resources. In this study, we present INDUS - a composition-based approach that incorporates the following novel features. First, INDUS discards the 'one genome-one composition' model adopted by existing compositional approaches. Second, INDUS uses 'compositional distance' information for identifying appropriate assignment levels. Third, INDUS incorporates steps that attempt to reduce biases due to database representation.

**Results:**

INDUS is able to rapidly classify sequences in both simulated and real metagenomic sequence data sets with classification efficiency significantly higher than existing composition-based approaches. Although the classification efficiency of INDUS is observed to be comparable to those by similarity-based approaches, the binning time (as compared to alignment based approaches) is 23-33 times lower.

**Conclusion:**

Given it's rapid execution time, and high levels of classification efficiency, INDUS is expected to be of immense interest to researchers working in metagenomics and microbial ecology.

**Availability:**

A web-server for the INDUS algorithm is available at http://metagenomics.atc.tcs.com/INDUS/

## Background

Microbial communities constitute the majority of life forms in any given environmental niche. In order to understand the structure of microbial communities, it is important to first characterize (in taxonomic and functional terms) the individual microbes that constitute these communities. Laboratory culture based approaches have been traditionally used for characterizing individual microbes. However, recent studies have revealed that almost 99% of microbes are difficult to culture in a laboratory [1]. The emerging field of metagenomics overcomes this limitation by adopting approaches that bypass the culturing step. In a typical metagenomic study, the entire genomic content of all microbes (irrespective of their culturability) in a given environmental sample is directly extracted, sequenced and characterized. In this process, millions of sequences of DNA fragments (originating from the genomes of diverse microbes) are obtained. Subsequently, computational methods are employed for predicting the taxonomic affiliation of these DNA sequences. This obtained information is collated for generating the taxonomic profile of a given microbial community.

Various approaches are available for obtaining the taxonomic affiliation of DNA sequences constituting a metagenomic sequence data set. These approaches can be broadly divided into two types, namely, similarity-based and composition-based. 'Similarity-based' approaches classify metagenomic sequences by comparing them with known sequences present in a reference database [2-5]. These comparisons are usually done using the BLAST algorithm [6]. The extent of similarity between metagenomic sequences and reference database sequences is inferred from the BLAST output. Query sequences are finally assigned to an organism/clade based on the pattern and quality of the generated BLAST hits. Similarity-based approaches are robust and are observed to have high binning accuracy since they infer taxonomic assignments by analyzing actual alignments obtained in the BLAST output. However, given the limited sequence information available in existing reference databases, majority of sequences in metagenomic data sets fail to obtain BLAST hits and are consequently categorized as 'unassigned'. Moreover, similarity-based approaches need enormous amount of time and computing resources for generating alignments of millions of metagenomic sequences with existing reference database sequences. On the other hand, 'Composition-based' approaches classify metagenomic sequences in the following manner. Compositional features (e.g. oligonucleotide frequency patterns) of known genomic sequences are first captured in the form of genome specific models. Amongst the composition-based classification methods, while TACOA [7] builds models based on the ratio of observed and expected frequencies of all possible tetra- and penta-nucleotides, Phylopythia [8] uses Support Vector Machines to capture patterns of oligonucleotide frequency distributions observed in available genome sequences. Similarly, a naive Bayesian approach is used by the NBC tool [9] for modelling the compositional properties of genomes. Subsequently, composition-based approaches score query sequences against the pre-computed genome specific models, and assign them to an organism/clade based on the pattern of scores obtained. Since the composition-based methods do not involve alignment of query sequences with reference database sequences, these methods are quicker as compared to similarity-based methods.

In spite of being rapid in execution, the existing composition-based approaches have three important limitations. First, none of the current composition-based approaches take into account the inherent non-uniform representation of the various taxonomic groups in existing reference databases. For example, approximately 60% of completely sequenced organisms (available in NCBI database) belong to phylum Proteobacteria. In contrast, very few organisms belonging to phyla like Fusobacteria (0.16%) and Chlorobi (0.16%) have been sequenced. This scenario tends to bias the scoring process of composition-based approaches towards models generated from genomes belonging to significantly over-represented phyla. Consequently, sequences originating from hitherto unknown organisms (especially belonging to phyla which are under-represented in existing databases) will tend to get incorrectly classified under taxa having over-represented taxonomic groups. This has a significant impact on the overall accuracy of taxonomic assignments.

The second limitation of the existing composition-based approaches pertains to the short lengths of the sequences being generated by next generation sequencing technologies. The typical length of metagenomic sequences is much below 1,000 base pairs. The statistical significance of oligonucleotide frequency values derived from such short sequences is thus low, and the taxonomic discrimination capability of binning algorithms using such low frequency values is also expected to be limited. Consequently, existing composition-based approaches have low binning specificity. In other words, a majority of metagenomic sequences are classified at non-specific taxonomic levels such as phylum or super-kingdom. Moreover, it is observed that existing classifiers like NBC tool or PhymmBL [10] only provide the score of all query sequences with pre-computed organism specific models. The task of interpreting these scores and appropriately reducing individual query assignments to corresponding higher taxonomic levels is left to the end user. Due to the absence of a linear correlation between the score and the correct taxonomic level of the predicted assignment, this interpretation is infeasible. This severely limits the utility of composition-based approaches in a metagenomic context, since a majority of sequences originate from hitherto unknown organisms/taxonomic groups, and it is necessary to classify each sequence at an appropriately higher taxonomic level (including genus, family, order, class and phylum, as well as, those lying at the tip of the taxonomy tree, i.e. 'root', cellular organisms and super-kingdom levels).

The third limitation of the existing composition-based approaches is due to the underlying premise of 'one genome-one composition model'. In other words, a single oligonucleotide usage pattern is assumed for any given genome. In practice, distinct trends of oligonucleotide usage are generally observed within a single genome [11]. For instance, in *Mycobacterium tuberculosis* and related species, approximately 4-10% of the genome codes for two different types of protein families, namely PE and PPE. Gene sequences coding for these proteins, although specific to the Mycobacterium genus, are highly repetitive and display an entirely different oligonucleotide composition as compared to the rest of the genome [12,13]. Assuming a single oligonucleotide composition model (based on a uniform oligonucleotide composition) for such genomes is thus expected to affect the accuracy and specificity of taxonomic classification of sequences originating from such compositionally distinct regions.

In spite of the above mentioned limitations, composition-based approaches are rapid in execution compared to similarity-based approaches, since the former approaches do not involve any alignment of individual query sequences to reference database sequences. They are thus well suited for binning metagenomic data sets (typically having millions of sequences) provided they exhibit binning efficiency comparable to that of similarity-based approaches.

As described above, binning algorithms are either similarity-based or composition-based. In contrast, the recently published SPHINX algorithm [14] utilizes the principles of both composition and similarity based binning algorithms. The SPHINX algorithm employs a unique (two-step) hybrid binning approach. In the first step, compositional characteristics of a given query sequence are utilized for identifying a subset of database sequences, that have compositional similarity with the query sequence. The second step involves aligning the query sequence with the identified subset of database sequences, analysing the aligned output, and finally assigning the query sequence to an appropriate taxon based on this analysis. The first step adopted by SPHINX (i.e reduction of search space using compositional features) is observed to reduce the binning time to a reasonable extent (without a significant loss in binning specificity/accuracy). However, given that the second step of SPHINX is still alignment based, the overall binning time is still dependant on the time taken for this alignment step.

In this study, we present INDUS - a novel algorithm which can taxonomically classify sequences at a rate which is significantly better compared to any of the binning approaches (including SPHINX) described above. Besides this, INDUS has binning accuracy and specificity comparable to alignment based approaches. Similar to the SPHINX algorithm, INDUS also adopts a two-step approach to binning. To some extent, the first step (i.e reduction of search space using compositional features) of both methods is similar. However, the INDUS algorithm incorporates several novel features in the second step, that make it an entirely alignment free (thereby drastically reducing the overall binning time) process. The second step also incorporates several new features that attempt to address various limitations of existing composition-based binning approaches.

## Results

### Algorithm

**Figure 1:**
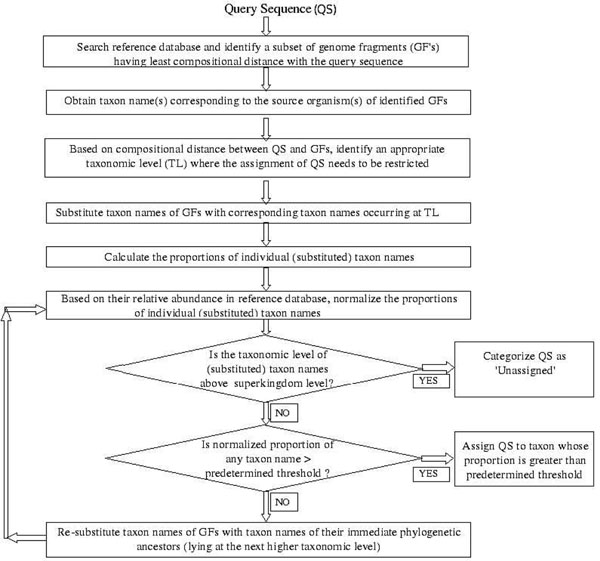
**Work-flow of the INDUS algorithm**. A schematic work-flow depicting the various steps followed by the INDUS algorithm for taxonomic assignment of query sequences.

The steps associated with the phylogenetic assignment of metagenomic sequences by INDUS are graphically depicted in Figure 1 and described below:

**Step A – identification of compositionally similar genome fragments:** The first step of INDUS involves identification of a subset of 'genome fragments' (generated from known prokaryotic genomes) whose composition is closest to that of the query sequence. For this purpose, known genome fragments (of length 1,000 bp) were generated by splitting 952 genome sequences downloaded from NCBI database (ftp://ftp.ncbi.nih.gov/genomes/Bacteria/). The compositional similarity between a given query DNA fragment and the generated genome fragments is calculated by finding the Manhattan distance between the tetra-nucleotide frequency vectors corresponding to the query sequence and each genome fragment. In order to overcome the enormous time required for comparing individual query vectors with each of the (2.6 million) genome fragment vectors, we utilized the SPHINX approach for reducing the search space [14]. In this approach, genome fragment vectors are initially clustered using the k-means clustering approach [15], and vectors corresponding to individual cluster centroids are stored. At run time, instead of performing 2.6 million comparisons, the distance of a given query sequence (represented in form of a vector) to each precomputed 'cluster centroid' is calculated. As in SPHINX, this step helps in first identifying a cluster having the least distance to the query vector. Till this step, INDUS and SPHINX adopt a similar strategy for reducing the initial search space. INDUS further identifies a subset of genome fragments within the compositionally closest cluster. For this purpose, the distance of the query vector to genomic fragments belonging only to the closest cluster is calculated and genome fragment(s) having a distance greater than or equal to 99% of the distance of the closest genomic fragment are retained. A subset of genome fragments closest in composition to a given query sequence are thus identified at the end of this step.

**Step B - identification of appropriate taxonomic levels of assignments:** The second step in the work-flow followed by INDUS algorithm involves identification of an appropriate taxonomic level (TL) at which the final assignment of the query sequence is to be restricted. Identification of this level is based on the premise/principle which has been adopted in earlier studies namely SOrt-ITEMS [4] and SPHINX [14]. A brief outline of the principle is as follows. Sequences from different organisms have diverged (evolved) from common ancestors. Consequently, as the level of taxonomic divergence between organisms increases, the compositional similarity between their sequences is expected to decrease progressively. It is implicit from this observation that the compositional similarity between two sequences, belonging to organisms that have diverged from relatively higher levels in the taxonomic tree, will be low. As a result, the 'distance' between tetra-nucleotide frequency vectors (a metric used for measuring compositional similarity) corresponding to sequences diverged from relatively higher levels in the taxonomic tree will be high. Assuming a uniform rate of evolution, the distance between any two sequences would thus indicate (to a large extent) the probable taxonomic level from where the sequences have diverged. It is logical to limit the assignment of the sequence at this taxonomic level, since this level would not only be conservative (thus avoiding false positives), but also the most specific level at which the assignment can be made. Keeping this principle in mind, using a large number of training sequences, patterns of distances were observed between sequences (diverged at various levels in the taxonomic tree), and corresponding thresholds were determined empirically. The detailed methodology used for obtaining threshold values (Table 1) is given in section A of Additional File 1.

**Table 1:** Range of thresholds for determining an appropriate taxonomic level of assignments (TL)

Lowest taxonomic level where the query sequence can be assigned	Distance range between query sequence and nearest genome fragment in reference database			
	
	Sanger (800 bp)	454-Titanium (400 bp)	454-Standard (250 bp)	454-GS20 (100 bp)
**Genus**	< 0.28	< 0.35	< 0.43	< 0.6
**Family**	0.28 – 0.32	0.35 – 0.41	0.43– 0.51	> 0.6
**Class**	> 0.32	> 0.41	> 0.51	

Range of distance values (between vectors corresponding to a query sequence and the closest genome fragment in reference database) to be used for determining an appropriate taxonomic level (TL) of assignment for a given query sequence.

Using the obtained threshold values, an appropriate taxonomic level (TL) is identified, where the assignment for each query sequence needs to be restricted. Subsequently, the taxonomic names of the identified subset of (compositionally closest) genomic fragments are replaced with corresponding taxon names that occur at TL.

**Step C - normalization of the proportions of individual taxa in the identified subset of genome fragments:** The proportions of individual taxa in the identified subset of genome fragments are first calculated. Normalization of these proportions is then carried out based on the relative abundance of these taxa in current reference databases using an empirically derived logarithmic normalization function given below:

where,

'N_i_' represents the normalized percentage of a particular taxon 'i' within the subset of genome fragments identified as closest to the given query sequence,

'P_i_' represents the percentage of a particular taxon 'i' within the subset of genome fragments identified as closest to the given query sequence,

'R_i_' represents the percentage of a particular taxon 'i' with respect to its representation in the reference database, and

'a' represents an integer with a value of 2 for query sequences generated using Sanger (read lengths around 800 bp), 454-Titanium (400 bp) and 454-Standard (250 bp) sequencing technologies and 0 for query sequences generated using 454-GS20 (100 bp) sequencing technology.

The methodology used to determine the optimal values of integer 'a' in the above logarithmic normalization function, for different query sequence lengths, is explained in section B of Additional File 1. A justification for using this logarithmic normalization function is provided in section C of Additional File 1.

**Step D - assignment of taxa to sequences:** The query sequence is associated to the taxon whose (normalized) proportion (within the set of closest genome fragments) exceeds a predetermined threshold value. The detailed methodology used for fixing the predetermined threshold value is given in section D of Additional File 1. If the normalized proportion of any of the taxa (within the set of closest genome fragments) does not exceed the predetermined threshold, INDUS reduces the taxon names to successively higher taxonomic levels. INDUS iteratively checks for a taxonomic level at which the proportion of a taxon (within the set of closest genome fragments) exceeds the predetermined threshold. If the normalized proportion does not exceeds the predetermined threshold even after reducing all taxon names (within the set of closest genome fragments) to the taxonomic level of super kingdom, the query sequence is categorized as unassigned.

### Validation

INDUS algorithm was validated by querying test sequences (constituting four simulated test data sets) against a reference database that was appropriately 'modified' to simulate realistic metagenomic scenarios. It is to be noted that these four simulated data sets and the modified reference database were identical to those used for evaluation of SPHINX algorithm [14]. A brief description of the four simulated data sets and the composition of the 'complete/modified' reference database are described below.

The four simulated test data sets, namely Sanger, 454-400, 454-250 and 454-100 were generated using MetaSim [16] software. As in SPHINX, each data set consisted of 35,000 query sequences originating from 35 taxonomically diverse prokaryotic genomes listed in Additional File 2. Sequences in these data sets simulated the sequencing lengths and error models of four commonly used sequencing technologies namely 'Sanger' (read length centered around 800 bp), '454-Titanium' (400 bp), '454-Standard' (250bp) and '454-GS20' (100 bp).

As in SPHINX, the 'complete reference database' (consisting of 2.6 million genome fragments from 952 prokaryotic genomes) was modified by completely removing fragments corresponding to 300 genomes. This resulted in a scenario wherein a majority of the sequences from each test data set originated from genomes, whose sequences are not represented in the reference database. Moreover, it was observed that the taxonomic level to which individual test sequences (originating from genomes of test organisms) were not represented in the reference database also varied. For instance, when (test sequences from) a given test organism was labeled as 'Class Unknown', genome fragments originating from all other genomes belonging to its class were removed from the reference database. Thus the 'modified reference database' along with the 'simulated test data sets' closely mimicked a real metagenomic scenario wherein majority of the sequences (in metagenomic data sets) are derived from unknown microbes. The representation status of each test data set organism with respect to modified database is also given in Additional File 2.

To maintain consistency of evaluation, parameters used in SPHINX were used for evaluating the performance of INDUS. Performance was characterized in terms of 'assignment accuracy' and 'assignment specificity'. Assignment accuracy demonstrates the ability of the algorithm to assign a given query sequence to its correct taxonomic lineage (till super kingdom level) irrespective of the phylogenetic level at which the assignment has been made. Assignment accuracy was determined by first grouping all assignments into 'Correct Assignments' and 'Wrong Assignments' and analyzing the respective percentages. On the other hand, assignment specificity indicates the ability to make correct assignments at specific taxonomic levels (species, genus, family, class, order and phylum) rather than at non-specific taxonomic levels (above the taxonomic level of phylum). Therefore, correct assignments were further grouped into 'Specific Assignments' and 'Non-specific Assignments' to evaluate the performance of INDUS with respect to assignment specificity. Results, both in terms of accuracy and specificity were compared with those obtained using composition-based i.e. TACOA [7], similarity-based i.e SOrt-ITEMS [4], MEGAN [2] and hybrid i.e. SPHINX [14] binning algorithms. To maintain consistency of evaluation, assignments of all five binning algorithms (used in the present study) were obtained with a ‘minimum bin size’ setting of 1 (i.e bins with a single sequence assignment were also considered for analysis).

### Comparison of execution time of taxonomic classification methods

The average computational time required by INDUS for taxonomic classification of 35,000 sequences (of each of the four validation data sets) was determined and compared with those by TACOA, SOrt-ITEMS, MEGAN and SPHINX. All calculations were performed on a desktop computer (Intel Xeon quad core processor, 4 GB RAM).

### Performance of INDUS on FAMeS (Fidelity of Analysis of Metagenomic Samples) metagenomic data sets

Given our limited knowledge of the true taxonomic composition of real metagenomes, it is difficult to evaluate the taxonomic classification efficiency of any binning algorithm using a real metagenomic data set. Keeping this in mind, the performance of INDUS was evaluated using three synthetically generated data sets [17], which nevertheless simulate true metagenomic scenarios. These data sets are considered as 'gold standard data sets' that can be used for benchmarking algorithms developed for analyzing metagenomics data [17]. Based on the level of taxonomic complexity, these data sets are referred to as simLC (low complexity), simMC (medium complexity), and simHC (high complexity). Taxonomic assignments of all sequences constituting these three data sets were obtained against two variants (referred to as 'complete' and 'partial') of the reference database. These variants are identical to those used earlier for evaluating validation data sets. While the 'complete' reference database consisted of genome fragments corresponding to 952 organisms, the 'modified' reference database' consisted of genome fragments from only 652 organisms. The latter database was created with the objective of replicating a realistic test scenario, wherein majority of the sequences (in the three metagenomic data sets) are derived from unknown microbes. The representation status of each test data set organism (in all three data sets) with respect to 'complete' and the 'modified' reference database is given in Additional File 3 and is explained below.

All three FAMeS data sets contained sequences sourced from 112 distinct genomes. Genomic fragments from these 112 genomes were also available in the 'complete' reference database. However, it is important to note that genomic fragments in the reference database are not exact copies of sequences constituting the three real metagenomic data sets. Besides containing typical sequencing errors (and stretches of low quality regions), the latter sequences originate from random positions in the respective genomes. In contrast to the complete reference database, the modified reference database had genomic fragments representing only 69 out of the 112 genomes constituting the FAMeS metagenomic data sets. Consequently, test sequences originating from 43 genomes had no representation (at various taxonomic levels) in the modified reference database. While 8 of these 43 genomes represented a 'species unknown' scenario, the 'genus unknown', 'family unknown', 'order unknown' and 'class unknown' scenarios were represented by 16, 14, 3 and 2 genomes, respectively. As done for the simulated test data sets, results of INDUS obtained with FAMeS data sets were compared with those obtained using TACOA, SOrt-ITEMS, MEGAN and SPHINX.

### **Performance of INDUS on a real metagenomic data set**

The performance of INDUS was further evaluated on the Sargasso sea data set [18]. This reasonably large (and taxonomically diverse) metagenomic data set containing 644,551 sequences was first evaluated using INDUS. Besides observing the binning time taken by INDUS for evaluating the complete data set, the cumulative percentage of assignments obtained at various taxonomic levels was noted down. Furthermore, a subset of 10000 sequences from the same data set, was analyzed using all 5 binning methods (INDUS, TACOA, SOrt-ITEMS, MEGAN and SPHINX). It should be noted that the same subset of sequences (referred to as Sargasso data set sample 1) were earlier used for evaluating the taxonomic binning efficiency of MEGAN [2] and SOrt-ITEMS [4]. Taxonomic assignments obtained by all five binning methods were compared at the level of phylum, class and order. This was done by first collapsing the obtained assignments (by a given method) at or below the desired taxonomic level of comparison and subsequently enumerating the same. The time taken for binning this subset of sequences was also noted down for all the methods.

### Validation results

#### Results with simulated test data sets

**Figure 2:**
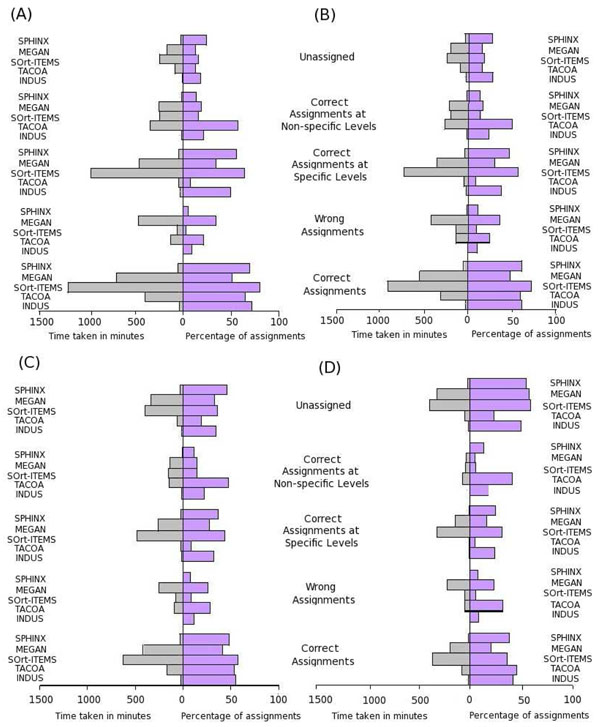
**Results of validation on simulated test data sets.** Graphical representation of the obtained pattern of assignments and the time taken by INDUS, TACOA, SOrt-ITEMS, MEGAN and SPHINX on the (A) Sanger, (B) 454-400 (C) 454-250 and (D) 454-100 test data sets.

Metagenomics data sets typically consist of millions of sequences, majority of which originate from hitherto unknown organisms. The interpretation of results was therefore done keeping the following aspects in mind. First is the time taken for binning. Given the huge size of typical metagenomic data sets, this aspect naturally becomes a very significant factor. Assignment accuracy and specificity are the second and third aspects. The objective was to interpret the obtained results (Figure 2 and Additional File 4) and identify a method that analyzes a million sequences within a few hours with reasonable levels of assignment accuracy and specificity.

With respect to the time taken for binning, results in section A of Additional File 4 indicate that INDUS is 23-33 times quicker than similarity-based methods (SOrt-ITEMS and MEGAN). Furthermore, INDUS is observed to be 6-12 times faster, even with respect to TACOA (a composition-based classifier). With respect to SPHINX (a composite method), INDUS is seen to be quicker by 1.5 to 1.6 times. In summary, these results indicate that amongst various available binning methods, INDUS is the quickest. Furthermore, our experiments with data sets of varying sizes (in terms of number of sequences and the length of sequences) also indicated that the execution time of INDUS increases linearly with the increase in the number of sequences in a given data set (Additional File 5). Given these results (with respect to binning time), it was logical to check the binning accuracy and specificity of INDUS. The overall pattern of results (Figure 2, Additional File 4) in this respect indicate that INDUS, in spite of being a composition-based method, is able to achieve comparable (and in some cases significantly better) results than other binning methods. Results with respect to binning accuracy and specificity are discussed below in detail.

Results obtained using all four methods (across all four data sets) indicate a positive correlation between the percentage of correct assignments and the length of the query sequences. For the 454-400, 454-250 and the Sanger test data sets, it is seen that assignment accuracy (with respect to the percentage of correct assignments) is observed to be more or less comparable for INDUS, TACOA and SPHINX. Though, the assignment accuracy of SOrt-ITEMS is observed to exceed the values obtained with all other methods, it is observed that SOrt-ITEMS requires enormous amounts of time (approximately 30-40 minutes per 1000 reads) for binning a given data set. At this rate, more than 10 days would be required by SOrt-ITEMS for binning even a small sized data set (500,000 reads each of length approximately 250 bp). Analysis of the same data set using INDUS is expected to be completed in less than 12 hours. Interestingly, for the 454-100 data set (having short sequences of length around 100 bp), the percentage of correct assignments by INDUS and TACOA (composition-based methods) is observed to be relatively higher compared to that obtained using similarity-based methods (i.e SOrt-ITEMS and MEGAN) as well as the composite method (i.e. SPHINX). However, it should be noted that a significant proportion of correct assignments by TACOA (41-57%) are at non-specific taxonomic levels (super kingdom or above) as compared to that by INDUS (18-24%), SOrt-ITEMS (5-16%), MEGAN (5-18%) and SPHINX (11-14%). With respect to 'wrong' assignments, results indicate that while the percentage of wrong assignments obtained with INDUS (10-12%) is significantly lower than that of TACOA (22-31%) and MEGAN (23-35%), it is slightly higher than SPHINX (7-12%) and SOrt-ITEMS (4 -10%).

The above results (correct and wrong assignments) capture the 'assignment accuracy' of each of the five evaluated methods. Overall, reasonable levels of assignment accuracy are seen to be obtained with INDUS (a composition-based method), SOrt-ITEMS (a similarity-based method) and SPHINX (a hybrid binning approach). The marginally higher misclassification rate of INDUS (as compared to that of SPHINX and SOrt-ITEMS) is possibly due to the following reason. Both SOrt-ITEMS and SPHINX algorithms employ BLAST, a relatively robust algorithm, for quantifying the similarity between query sequences and reference database sequences. However, the enormous binning time (and the compute resources) needed by similarity-based methods limits their practical utility in resource poor settings.

Correct assignments were further categorized into 'specific', 'non-specific' and 'unassigned' categories. The percentage of test sequences assigned to these categories would help in comparing the 'assignment specificity' of each algorithm. As expected, it is observed that assignment specificity increases with increasing length of query sequences. Furthermore, results indicate that the average assignment specificity of INDUS (35.9%) is significantly higher than that of TACOA (6.9%) and MEGAN (26.5%), and lower than that of SOrt-ITEMS (48.8%) and SPHINX (40.7%). Results also indicate that the percentage of assignments made by INDUS (21.3%) at non-specific levels is significantly lower than that by TACOA (48.7%) and slightly higher than that by SOrt-ITEMS (12.3%), MEGAN (13.6%) and SPHINX (13.3%).

The above results indicate that the assignment accuracy of INDUS is not at the cost of assignment specificity. With respect to the percentage of sequences categorized as 'unassigned', results (with all four test data sets) indicate a negative correlation between the percentage of 'unassigned' sequences and the length of the query sequences (irrespective of the method used). As in the case of correct assignments, the percentage of sequences categorized as 'unassigned' by INDUS (19-49%) is observed to be comparable to that obtained using similarity-based methods i.e. SOrt-ITEMS (16-59%), MEGAN (13-57%), and the hybrid method SPHINX (24-55%). The corresponding percentage of unassigned sequences by TACOA (13-23%) appears to be lower than the other three methods. However, as mentioned previously, 41-57% of assignments made by TACOA are at non-specific levels. Such non-specific assignments have little practical significance.

#### **Results with FAMeS metagenomic data sets**

For each FAMeS metagenomic data set, the percentage of assignments (in various categories) obtained by INDUS, TACOA, SOrt-ITEMS, MEGAN and SPHINX is given in Additional File 6. A summary of these results is given in Table 2. Results indicate that 79-93% of sequences are correctly assigned by INDUS in both complete and modified reference database scenarios. These values are comparable to that obtained using SOrt-ITEMS (77-95%), SPHINX (78-93%) and MEGAN (70-95%), and are relatively better compared to TACOA (67-83%). These results indicate the robustness of the overall INDUS classification approach and the ability of INDUS to generate results comparable to similarity-based approaches. With respect to assignment specificity, it is observed that SOrt-ITEMS has the highest average value of specificity (78-94%) in both database scenarios. The average assignment specificity of INDUS (67-81%), MEGAN (66-92%) and SPHINX (71-81%) is observed to significantly exceed that obtained by TACOA (21-22%). The overall misclassification rate (irrespective of reference database status) of INDUS is significantly low and ranges between 2-6%. This reaffirms that the assignment specificity obtained by INDUS is not at the cost of assignment accuracy (as observed for MEGAN). The marginally high misclassification rate of INDUS with the simLC data set (7.3%) as compared to the simMC data set (4.51%) and the simHC data set (5.8%) is due to the following reason. Approximately 37.3% of sequences in the simLC data set have a 'genus unknown' status with respect to the modified reference database. This is significantly higher than that compared to 26.1% in the simMC and 14.8% in the simHC data set (Additional File 3). Overall, all the above results indicate that the influence of taxonomic complexity on the assignment accuracy of INDUS is minimal. This is expected given that INDUS (as well as other existing binning algorithms used in the present study) independently process each query sequence. The taxonomic assignment obtained for a given query sequence is independent of the taxonomic complexity of the sample to which the sequence belongs. In line with this observation, results indicate an absence of correlation between algorithmic performance and taxonomic complexity of the sample.

**Table 2:** Results of validation on FAMeS Data sets

FAMeS Data set	Taxonomic assignment category	Results with complete database					Results with modified database				
	
		INDUS	TACOA	SOrT-ITEMS	MEGAN	SPHINX	INDUS	TACOA	SOrT-ITEMS	MEGAN	SPHINX
SimLC (96732)	Correct	89.6	79.3	94.9	95	93.1	81.6	74.4	78.1	72.6	83.7
	Wrong	2.6	9	2	2.7	2.8	7.1	13.9	9.2	23.6	11.5
	Specific	81.3	24.4	94.9	93.9	82.8	66.7	21	78.1	65	71
	Non-specific	8.3	54.9	0	1.1	10.3	14.9	53.5	0	7.6	12.7
	Unassigned	7.9	11.6	3.1	2.3	4.1	11.4	11.6	12.6	3.7	4.8

SimMC (113373)	Correct	92.8	82.5	93.7	94	92.6	86	79	79.1	69.9	81.8
	Wrong	1	7.3	3.1	3.5	3.6	4.6	10.8	9.1	26.6	13.5
	Specific	84.1	23.7	93.7	93.1	81.1	71.9	23	79.1	63.2	70.1
	Non-specific	8.7	58.8	0	1	11.5	14.1	56	0	6.7	11.6
	Unassigned	6.2	10.2	3.2	2.4	3.8	9.4	10.2	11.8	3.5	4.7

SimHC (115592)	Correct	89.6	72.1	92	91.9	86	78.9	67	76.6	77.9	77.6
	Wrong	1.9	11.4	3.6	4.9	7.1	5.7	16	9.5	17.6	10.1
	Specific	76.4	18.7	92	90.3	79.6	63.4	18.2	76.6	69.5	71.2
	Non-specific	13.2	53.4	0	1.5	6.4	15.5	48.8	0	8.4	6.4
	Unassigned	8.6	16.5	4.3	3.2	6.9	15.5	17	13.9	4.5	12.3

Average for FAMeS data sets	Correct	90.6	78	93.5	93.6	90.6	82.2	73.5	77.9	73.5	81
	Wrong	1.8	9.3	2.9	3.7	4.5	5.8	13.6	9.3	22.6	11.7
	Specific	80.6	22.3	93.5	92.4	81.2	67.3	20.7	77.9	65.9	70.8
	Non-specific	10.1	55.7	0	1.2	9.4	14.8	52.8	0	7.6	10.2
	Unassigned	7.6	12.8	3.5	2.6	4.9	12.1	12.9	12.8	3.9	7.3

Summary of the results obtained with the FAMeS metagenomic data sets. The complete and modified reference database contained genome fragments from 952 and 652 prokaryotic genomes respectively. The number of sequences in each data set is indicated in parenthesis.

#### **Results with real metagenomic data set**

The summarized results obtained with INDUS for the Sargasso sea metagenomic data set [18] are provided in Table 3. INDUS was able to assign 545277 out of 644551 sequences (approximately 85%) constituting this data set. Around 78% of these assignments (429056 out of 545277) were made at specific taxonomic levels (i.e phylum and below). The cumulative number of assignments at phylum, class and order levels is indicated in Table 3. Besides assigning approximately 4% of sequences to phylum Cyanobacteria (normally expected in sea samples), it is observed that INDUS assigns a high proportion of sequences (approximately 60%) to various taxa under phylum Proteobacteria. These results are in concordance with earlier reported marker gene based analyses of this data set [18]. However, as previously mentioned, given our limited knowledge of the true taxonomic composition of real metagenomes, it is difficult to comment on the classification accuracy of any binning algorithm using result of analysis from a real metagenomic data set. Furthermore, it is significant to note that the total time taken by INDUS (on a modest desktop with 2GB RAM, 2.33 GHz dual-core processor) for analysing the 644,551 sequences of this real metagenomic data set was approximately 36 hours. Using the same desktop, an estimated 2-3 weeks would be required by existing similarity-based methods for analysing the same data set.

**Table 3:** Validation results of INDUS with Sargasso sea metagenomic data set.

**Order** (308709)			**Class** (361653)	
Burkholderiales	31.02		Betaproteobacteria	33.93
Alteromonadales	10.64		Gammaproteobacteria	20.07
Prochlorales	1.81		Alphaproteobacteria	0.9
Aeromonadales	1.22		Bacilli	0.34
Chroococcales	1.06		Clostridia	0.29
Enterobacteriales	0.53		Actinobacteria (class)	0.26
Pseudomonadales	0.52		Mollicutes	0.25
Rhizobiales	0.26		Spirochaetes (class)	0.03
Clostridiales	0.21		Deltaproteobacteria	0.02
Actinomycetales	0.11		Epsilonproteobacteria	0.02
Rickettsiales	0.11			
	
Mycoplasmatales	0.09		**Phylum** (429056)	
Bacillales	0.07		Proteobacteria	60.98
Xanthomonadales	0.06		Cyanobacteria	3.67
Lactobacillales	0.04		Firmicutes	1.15
Nitrosopumilales	0.04		Actinobacteria	0.3
Spirochaetales	0.03		Tenericutes	0.29
Thiotrichales	0.02		Thaumarchaeota	0.06
Campylobacterales	0.02		Spirochaetes	0.04
Rhodobacterales	0.02		Bacteroidetes	0.02
Vibrionales	0.02		Euryarchaeota	0.02
-	-		Thermotogae	0.02
-	-		Planctomycetes	0.02

Taxonomic profile, indicating the cumulative percentage of sequences assigned at various taxonomic levels. The cumulative number of sequences assigned at each taxonomic level is indicated in parenthesis.

Table 4 shows a comparison of the performance of INDUS, TACOA, SOrt-ITEMS, MEGAN and SPHINX on the Sargasso sea sample 1 data set. Results in Table 4 indicate that all five binning methods assign approximately 81-90% of the 10000 sampled sequences constituting this data set. As observed in the results obtained with the simulated test data sets, INDUS (58%) is observed to have more specific level assignments (phylum and below) as compared to SPHINX (53%) and TACOA (35%). SOrt-ITEMS and MEGAN (both similarity-based methods) are observed to assign more than 80% of sequences at phylum or below levels. However, with respect to the overall binning time, INDUS (13 minutes) is observed to be 25 times faster than SOrt-ITEMS (347 minutes) and MEGAN (321 minutes), and approximately 1.7 times faster than SPHINX (23 minutes). Furthermore, results at finer taxonomic levels (class and order) indicate that all methods (except TACOA) assign more or less similar proportion of sequences to taxa under phylum Proteobacteria. However, in contrast to SOrt-ITEMS and MEGAN, it is observed that INDUS and SPHINX do not assign any sequences to class Alphaproteobacteria. Our analysis revealed that more than 95% of these sequences (assigned by SOrt-ITEMS and MEGAN to Alphaproteobacteria) were assigned by INDUS and SPHINX to relatively higher taxonomic levels (either to proteobacteria or bacteria) and not to other unrelated taxa.

**Table 4:** Comparison of results of INDUS with other binning methods for Sargasso sea sample 1* metagenomic data set.

Binning method	Total number of sequences	Time taken for analysis (minutes)	Total number of sequences assigned	Cumulative number of sequences assigned at different taxonomic levels		
	
				Phylum	Class	Order
INDUS	10000	13	8167	5793	4416	3748
TACOA	10000	180	8870	3518	2739	2545
SOrt-ITEMS	10000	347	8528	8173	6921	5506
MEGAN	10000	321	8866	8417	7559	7461
SPHINX	10000	23	9116	5346	3702	2726



**Taxonomic level**	**Taxon name**	Percentage** of sequences assigned				
	
		**INDUS**	**TACOA**	**SOrt-ITEMS**	**MEGAN**	**SPHINX**

**Order**	Burkholderiales	22.79	16.75	25.6	28.63	20.31
	Alteromonadales	12.81	5.57	17.24	18.65	5.57
	Rickettsiales	-	-	5.58	12.78	-
	Prochlorales	1.88	-	3.01	2.94	-
	Enterobacteriales	-	1.75	-	-	-
**Class**	Betaproteobacteria	24.31	16.76	28.2	28.89	20.31
	Gammaproteobacteria	19.85	9.48	22.91	24.6	16.71
	Alphaproteobacteria	-	-	15.55	18.41	-
	Flavobacteria	-	-	-	2.12	-
**Phylum**	Proteobacteria	52.54	32.1	73.6	75.71	47.78
	Cyanobacteria	3.73	0.53	4.84	4.83	-
	Firmicutes	1.66	1.25	0.27	0.25	4.17
	Bacteroidetes	-	0.01	1.97	2.19	-
	Tenericutes	-	0.47	-	-	1.51

The cumulative percentage of sequences assigned by INDUS, TACOA, SOrt-ITEMS, MEGAN and SPHINX at order, class and phylum levels

* Sample 1 refers to the subset of 10000 reads from the Sargasso sea data set [18] earlier analysed using MEGAN [2] and SOrt-ITEMS [4]

** Percentages shown are with respect to the total number of sequences (i.e 10000) in the Sample 1 data set. Only those taxa are shown for which at least one of the methods assigned a minimum of 1.5% of the sequences in the data set.

## Discussion

The basic principle of existing composition-based approaches, i.e. 'the magnitude of compositional similarity is indicative of phylogenetic proximity', is the premise on which the INDUS algorithm is built. However, INDUS incorporates major modifications in its classification approach in order to overcome the following known limitations of existing composition-based approaches.

First, INDUS discards an important assumption used by existing composition-based approaches, namely, 'one genome-one composition' model. This model is based on the assumption that any fragment derived from a given genome is, more or less, a compositional fractal of that genome. By first identifying a subset of 'genome fragments' (and not genomes) whose composition is closest to that of the query sequence, INDUS algorithm ensures elimination of this fractal bias. Moreover, in this step, comparison of compositional properties is fair since it is performed between roughly equal sized fragments. This is better than previous approaches, wherein, comparisons were performed between compositional properties of short query sequences (less than 1,000 bp) and compositional models generated using whole genomes generally having lengths in excess of a million base pairs.

Second, INDUS uses compositional similarity as a metric to identify an appropriate taxonomic level (TL) of assignment for every query sequence. This step helps INDUS in achieving significantly low misclassification rates. The TL indicates a 'safe' (yet specific to the extent possible) taxonomic level at which the subsequent phylogenetic assignment process (based on taxonomic convergence) can commence. Identifying an appropriate taxonomic assignment level is important given that a majority of sequences in a typical metagenome originate from hitherto unknown organisms having little or no representation in existing reference databases. It is logical to assign such sequences at appropriately higher taxonomic levels.

Third, the phylogenetic assignment process (based on taxonomic convergence) used by INDUS helps in imparting high levels of accuracy. Considering a subset of fragments for every query sequence (instead of just one closest fragment), and subsequently identifying a taxonomic level (at or above TL) where these fragments achieve taxonomic convergence helps in further enhancing the accuracy of the entire procedure. Assignment of each (novel) sequence directly at an appropriately higher taxonomic level eliminates the need for the end user to interpret any scores and globally reduce assignments at a single taxonomic level (as required for results generated using classifiers such as Phymm, NBC classifier, etc.). The output of INDUS thus helps one in obtaining an unambiguous picture of the taxonomic profile of any given metagenome.

Fourth, existing reference databases are dominated with sequences corresponding to organisms/taxonomic clades that are amenable to culture techniques. Besides, reference databases are also biased with sequences belonging to specific organisms/taxonomic clades that have high scientific/commercial utility. For a query sequence originating from a sparsely represented taxon, such representation bias may get reflected in the identified subset of compositionally closest genome fragments. The identified subset may therefore contain genomic fragments from phylogenetically unrelated taxa with high representation in the reference database. In this scenario, the normalization function used by INDUS attempts to down-weigh the proportions of abundant taxa in order to achieve accurate assignments. At the same time, INDUS ensures that the abundant taxa proportions are not reduced to inappropriately low levels in scenarios wherein the query sequences originate from taxonomic clades having a high database representation. The normalization function used in INDUS therefore works optimally with sequences from any organism, irrespective of its proportion in existing reference databases.

In addition to overcoming the limitations of existing composition-based approaches, the quick execution time of INDUS confers it a great advantage over other binning methods. INDUS is able to analyze approximately 1000-1500 reads per minute. This rate of execution is significantly high compared to the execution rate (around 25-30 reads per minute) of similarity-based approaches such as SOrt-ITEMS or MEGAN. An estimate of binning time needed by INDUS (and other binning approaches) for analyzing real metagenomic data sets [18-23] is provided in Table 5. Results in this table reaffirm the utility of INDUS in experimental labs having limited access to huge computational resources. For example, it is observed that the analysing the 7,521,215 sequences (average length around 800 bp) constituting the Global Ocean Sampling Expedition Microbial Metagenomic data sets [18-20] using INDUS (on a desktop having an Intel Xeon-Quad core processor and 4 GB RAM) would have taken approximately 7 days. In contrast, analyzing the same data set using TACOA, SOrt-ITEMS, MEGAN and SPHINX would have taken approximately 90, 221, 200 and 11 days respectively. Analysis of even a smaller data set, for e.g. 1,744,283 sequences (with average read length around 100 bp) of the mouse gut metagenome [21] using SOrt-ITEMS or MEGAN would require at least a month for completing the analysis. In contrast, INDUS would complete the taxonomic analysis of this data set within a day. It is to be emphasized here that INDUS is able to achieve this binning rate without a significant loss of binning accuracy and specificity.

**Table 5:** Estimates of time required for taxonomic binning of some real metagenomic data sets

Metagenome	Total number of sequences	Sequence length range	Approximate estimate of time (in minutes) need for binning					Reference (s)
		
			INDUS	TACOA	SOrt-ITEMS	MEGAN	SPHINX	
Global Ocean Survey	7521215	~800bp	10530 (~ 7 days)	129580 (~90 days)	319330 (~221 days)	287095 (199 days)	15901 (11 days)	[18-20]
Lean and obese mouse metagenome	1744283	~100bp	1544 (~ 1 day)	8771 (6 days)	52097 (36 days)	48390 (33 days)	2093 (1.5 days)	[21]
Malnourished child metagenome	1496170	~250bp - 400bp	1795 (1.2 days)	17526 (12 days)	51297 (~36 days)	44885 (~31 days)	2308 (1.6 days)	[22]
Acid Mine Drainage	180713	~800bp	252	3113	7672	6898	382	[23]

Approximate time (in minutes) estimated to be taken by INDUS, TACOA, SOrt-ITEMS, MEGAN and SPHINX for binning some of the real metagenomic data sets (on a desktop with an Intel Xeon-Quad core processor and 4 GB RAM)

In spite of the advantages described above, one of the computational challenges for the INDUS approach (and also for 'one genome-one composition model based approaches) is the accurate taxonomic classification of metagenomic sequences originating from horizontally transferred (HGT) regions. Sequences originating from HGT regions generally have an unusual composition as compared to the sequences originating from the rest of the genome. The pattern of taxonomic assignment of such sequences depends to a large extent on their origin as well as the presence/absence of the donor/recipient genomes in the reference database. Additional File 7 summarizes the probable assignment patterns for such sequences. However, it is to be noted that for such cases, the interpretation of taxonomic assignment (as correct/incorrect) is subject to debate. The pattern of assignments (with respect to assignment specificity) is also likely to change as more and more genomes are sequenced and added to the reference database. Moreover, given that the proportion of HGT regions in a majority of known genomes is found to be around 10% [24], the impact of such assignments on the overall accuracy and specificity of INDUS (and other binning algorithms) is expected to be minimal.

A careful examination of the taxonomic classification methodology of INDUS indicates procedural similarities (albeit at a generic level) with that adopted by SPHINX. Additional File 8 summarizes these high level similarities. However, it is to be noted that the finer methodology and the similarity metrics used by both methods are significantly different. Though both methods utilize compositional features for reduction of search space, the procedural similarity is limited only till the identification of a cluster that shows composition similarity with the composition of the query sequence. While INDUS proceeds to further identify a 'subset' of 'compositionally closest' genome fragments within the identified cluster, SPHINX performs a 'alignment based search' of the query with 'all' sequences belonging to this cluster. Furthermore, INDUS in its subsequent steps, utilizes 'compositional distance' and 'taxonomic convergence' as the criteria for the final assignment of the query sequence. SPHINX, in contrast, relies on sequence alignment and on the generated alignment parameters. Despite these differences in methodology, INDUS and SPHINX display similar levels of binning efficiency. This is interesting in itself, as it indicates that 'sequence composition' as a feature for binning can be as effective as 'sequence similarity'. Moreover, it should be noted that the overall execution time of composition-based algorithms is significantly less than pure sequence similarity-based methods. This assumes significance given that metagenomic sequence data sets typically contain millions of sequences and using similarity-based methods for taxonomic assignment will require enormous time and compute power for analysis.

## Conclusions

The overall taxonomic assignment efficiency of INDUS is observed to be comparable to that of similarity-based methods and considerably superior to composition-based methods. At the same time the processing times required by INDUS for taxonomic classification is significantly low, a characteristic of composition-based methods. Moreover, the high assignment accuracy and assignment specificity of INDUS with metagenomic data sets (simLC, simMC and simLC having varying levels of taxonomic complexity) in database scenarios simulating real metagenomic conditions, reaffirm the utility and applicability of INDUS for performing a taxonomic classification of real-world metagenomic data sets.

## Competing interests

The authors declare that they have no competing interests.

## Authors' contributions

MHM, TSG, NKS and SSM have conceived the idea and designed the detailed methodology. NKS, TSG, RMR, CVSKR have implemented the algorithm. MHM, TSG, RMR created validation data sets and carried out detailed validation and testing of the algorithm. MHM, RMR, TSG and SSM have analyzed the data and finally drafted the complete paper.

## Supplementary Material

Additional file 1 **Threshold determination and parameter optimization** A document describing the following: a. The methodology used for obtaining distance threshold values for identifying an appropriate taxonomic level of assignment. b. The methodology adopted for characterization of parameters for the logarithmic normalization. c. Validation of the efficiency of normalization procedure. d. The methodology for the assignment of taxa to query sequences. Click here for file

Additional file 2 **List of organisms constituting the four test data sets** A document containing the list constituting the four simulated test data sets and their status with respect to the modified reference database. Click here for file

Additional file 3 **List of organisms constituting the FAMeS data sets.** A document containing the list constituting the simHC, simMC and the simLC data sets and their status with respect to the modified reference database. Click here for file

Additional File 4 **Detailed results of validation on the simulated test data sets** A document summarizing the pattern of taxonomic assignments and the time taken by INDUS, TACOA, SOrt-ITEMS, MEGAN and SPHINX on the four simulated test data sets. Click here for file

Additional file 5 **Time performance of the INDUS algorithm** A document containing the time taken by INDUS for binning 10000, 20000, 100000 and 500000 sequences. Click here for file

Additional File 6 **Detailed results of validation on the FAMeS data sets** A document containing the summarized results of (A) INDUS (B) TACOA (C)SOrt-ITEMS (D) MEGAN and (E) SPHINX obtained for the simLC, simMC and simHC data sets. Click here for file

Additional File 7 **Probable taxonomic assignment patterns for Horizontal Gene Transfer (HGT) regions** A document summarizing the probable pattern of taxonomic assignment (obtained using INDUS and a 'one-genome-one-composition' model based method) for sequences that originate from genomic regions involved in lateral gene transfer events. Click here for file

Additional File 8 **Similarities/dissimilarities between INDUS and SPHINX** A document summarizing the similarities/dissimilarities in the overall taxonomic assignment procedure adopted by INDUS and SPHINX. Click here for file
