# Supplementary material for: INDUS - a composition-based approach for rapid and accurate taxonomic classification of metagenomic sequences
Source: BMC Genomics. 2011 Nov 30;12(Suppl 3):S4. doi: 10.1186/1471-2164-12-S3-S4 (PMC3333187; doi:10.1186/1471-2164-12-S3-S4)
Supplement: Additional file 1 — Threshold determination and parameter optimization A document describing the following: a. The methodology used for obtaining distance threshold values for identifying an appropriate taxonomic level of assignment. b. The methodology adopted for characterization of parameters for the logarithmic normalization. c. Validation of the efficiency of normalization procedure. d. The methodology for the assignment of taxa to query sequences. [file 1471-2164-12-S3-S4-S1.pdf]

SUPPLEMENTARY MATERIAL

SECTION A

METHODOLOGY USED FOR OBTAINING DISTANCE THRESHOLD VALUES USED FOR IDENTIFICATION OF AN APPROPRIATE 'TAXONOMIC LEVEL' (TL) OF ASSIGNMENT

To determine the distance threshold values appropriate for sequences obtained using various variants of sequencing technologies, i.e Sanger (sequence length around 800 bp), 454-Titanium (400 bp), 454-Standard (250 bp) and 454-GS20 (100 bp), four sets of simulated sequences corresponding to these four sequencing technologies were generated using MetaSim software (Huson *et a*l., 2008) and employed as training data sets. Each set (55,000 sequences) contained sequences generated from 55 organisms belonging to 55 unique prokaryotic orders. The details of 55 organisms constituting these data sets are tabulated in Table S1. A reference database consisting of known genome fragments (of length 1,000 bp) was generated by splitting 952 genome sequences downloaded from NCBI database (ftp://ftp.ncbi.nih.gov/genomes/Bacteria/all.fna.tar.gz). In addition to the complete reference database, five 'modified' variants of the reference database were created by progressively removing sequences corresponding to genus, family, order, class and phylum of the source organisms of the sequences. The following procedure was subsequently performed for sequences within each training data set. Using Manhattan distance between the tetra-nucleotide frequency vectors as a similarity measure, the closest reference genomic fragments (in each of the six reference databases) was identified for each query sequence of a training data set. These query sequence and its closest genomic fragment (hereafter referred to as query-hit pair) generated against each database were written to separate output files. Thus, six different outputs were generated. These output files were further processed as follows. If the taxon name corresponding to the source organism of the nearest genomic fragment belonged to the same genus as the source organism of the query sequence, the query-hit pair was tagged as 'diverged from genus'. Similarly, if the taxon name corresponding to the source organism of the nearest genomic fragment matched with the query sequences' source organism at either the family or order or class or phylum or super kingdom levels, the query-hit pair was tagged as 'diverged from genus', 'diverged from family', 'diverged from order', 'diverged from class', 'diverged from phylum', and 'diverged from super kingdom' respectively. Query-hit pairs having similar tags were grouped together. The distribution of distances of query-hit pairs within each group was plotted (Figures S1A-D). From these plots, the distance thresholds for restricting the assignment of query sequences (to appropriate taxonomic levels) applicable for Sanger, 454-Titanium, 454-Standard, and 454-GS20 sequences were identified. The identified taxonomic levels (inferred from Figures S1A-D) represent the TLs. For example, if the distance of the nearest genomic fragment to a query sequence (of size 800 bp) is 0.28 (Figure S1A), TL is identified as family level. Therefore, the taxonomic names of the identified subset of (compositionally nearest) genomic fragments are first substituted with corresponding taxon names that occur at family level. The proportions of substituted taxa in the identified subset of genome fragments are then calculated and are normalized based on the relative abundance of these taxa in current reference databases. In the final step, the process of identifying whether the (normalized) proportion of a taxon exceeds a predetermined threshold value thus begins from the level of family. If the normalized proportion of any of the taxa (within the set of closest genome fragments) does not exceed the distance threshold at family level, INDUS iterates the final step after reducing the taxon names to successively higher taxonomic levels (i.e order, class, phylum, etc).

Results obtained with data set containing sequences of length ~ 800 bp (Sanger)

Based on the observation (Figure S1A) that majority of query sequences (>69%) having a distance value < 0.28 were tagged as 'diverged from genus', a distance threshold < 0.28 was used for considering the assignment of sequences from genus levels. In the distance range 0.28-0.32, it was seen that approximately 75% of sequences were tagged either as 'diverged from family' or 'diverged from order'. Based on these observations, the assignment of sequences having the nearest genomic fragment (in reference database) at a distance between 0.28-0.32 was considered from family level. Using similar reasoning, assignment of sequences having the nearest genomic fragment (in reference database) at a distance greater than 0.32 was considered from class level.

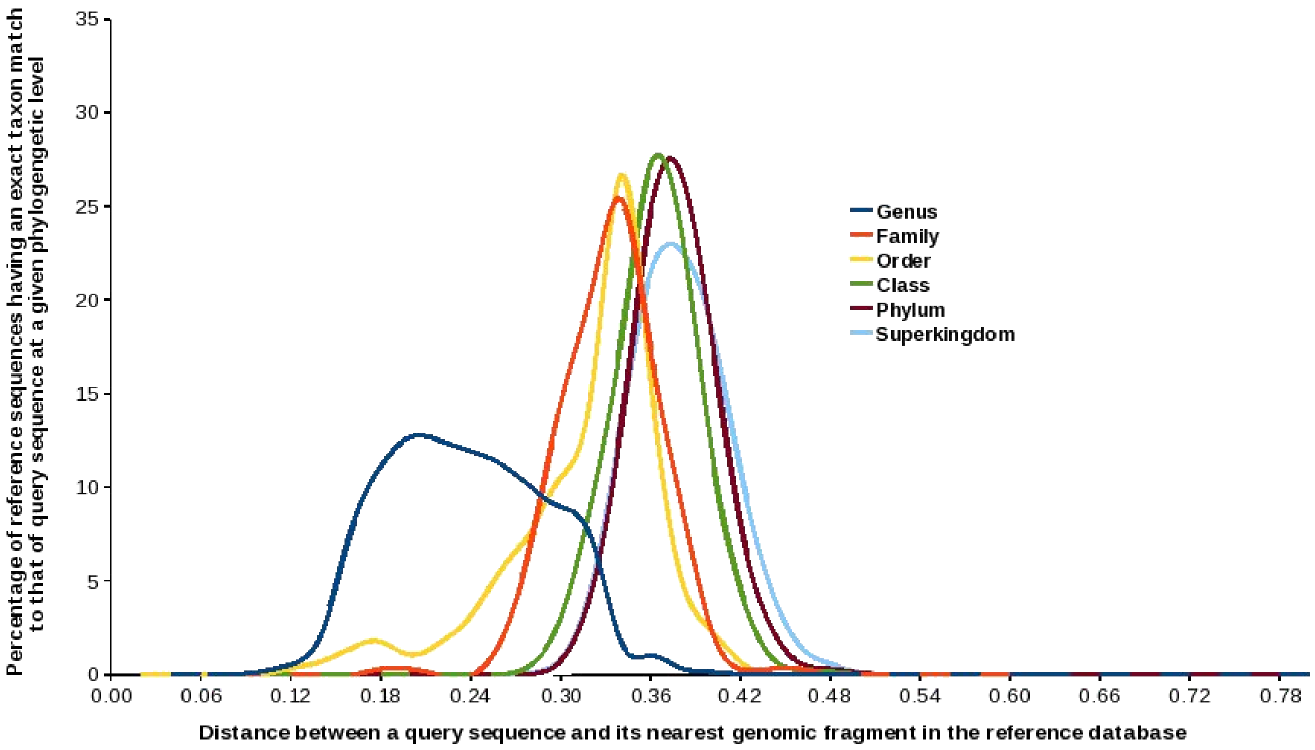

Figure S1A: Plot showing the percentage of sequences in the simulated Sanger data set in different distance ranges. Sequences tagged as 'diverged from genus', 'diverged from family', 'diverged from order', 'diverged from class', 'diverged from phylum' and 'diverged from super kingdom' are abbreviated as 'Genus', 'Family', 'Order', 'Class', 'Phylum' and 'Super kingdom', respectively.

Results obtained with data set containing sequences of length ~ 400 bp (454-Titanium)

Based on the observation (Figure S1B) that majority of query sequences (>59%) having a distance value < 0.35 were tagged as 'diverged from genus', a distance threshold < 0.35 was used for considering the assignment of sequences from genus levels. In the distance range 0.35-0.4, it was seen that approximately 62% of sequences were tagged either as 'diverged from family' or 'diverged from order'. Based on these observations, the assignment of sequences having the nearest genomic fragment (in reference database) at a distance between 0.35-0.4 was considered from family level. Using similar reasoning, assignment of sequences having the nearest genomic fragment (in reference database) at a distance greater than 0.40 was considered from class level.

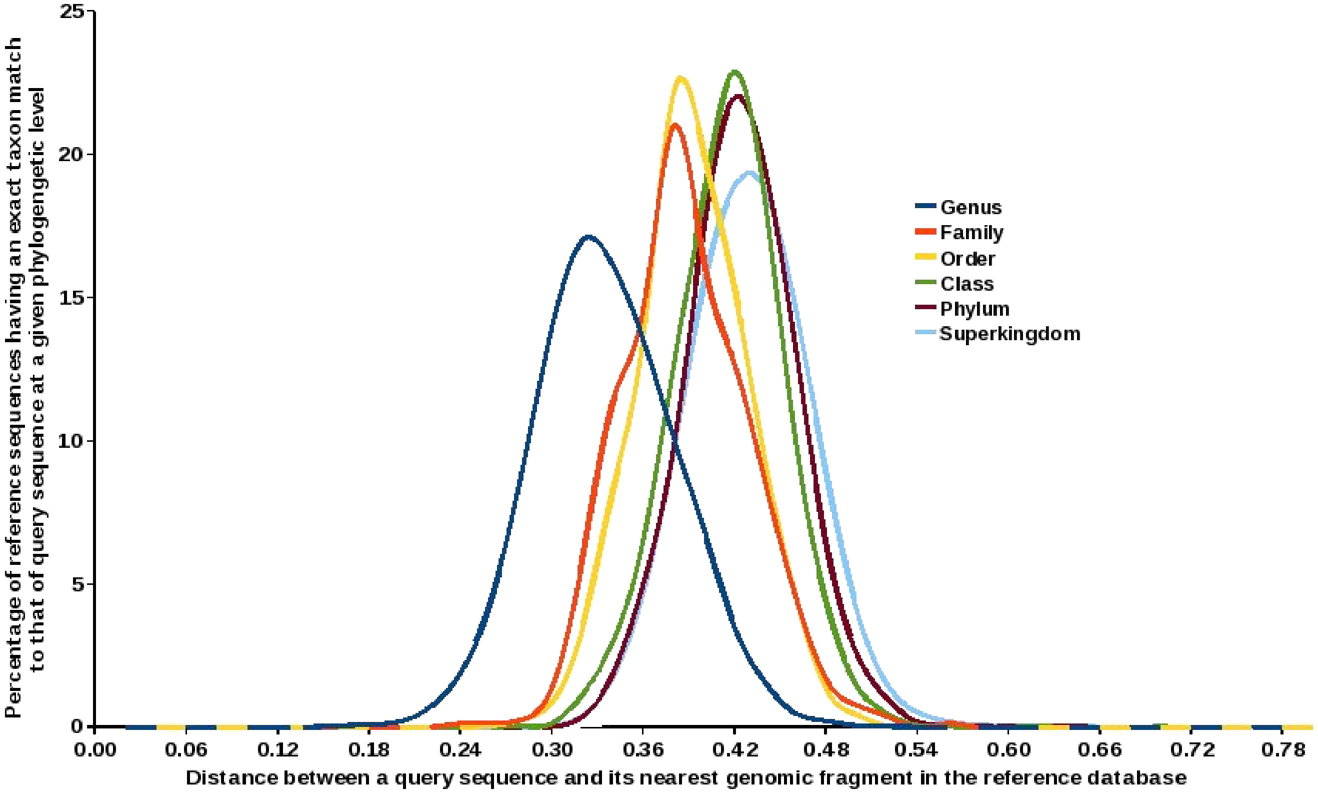

Figure S1B: Plot showing the percentage of sequences in the simulated 454-400 data set in different distance ranges. Sequences tagged as 'diverged from genus', 'diverged from family', 'diverged from order', 'diverged from class', 'diverged from phylum' and 'diverged from superkingdom' are abbreviated as 'Genus', 'Family', 'Order', 'Class', 'Phylum' and 'Super kingdom', respectively.

Results obtained with data set containing sequences of length ~ 250 bp (454-Standard)

Based on the observation (Figure S1C) that majority of query sequences (>60%) having a distance value < 0.43 were tagged as 'diverged from genus', a distance threshold < 0.43 was used for considering the assignment of sequences from genus levels. In the distance range 0.43-0.51, it was seen that approximately 62-69% of sequences were tagged either as 'diverged from family' or 'diverged from order'. Based on these observations, the assignment of sequences having the nearest genomic fragment (in reference database) at a distance between 0.43-0.51 was considered from family level. Using similar reasoning, assignment of sequences having the nearest genomic fragment (in reference database) at a distance greater than 0.51 was considered from class level.

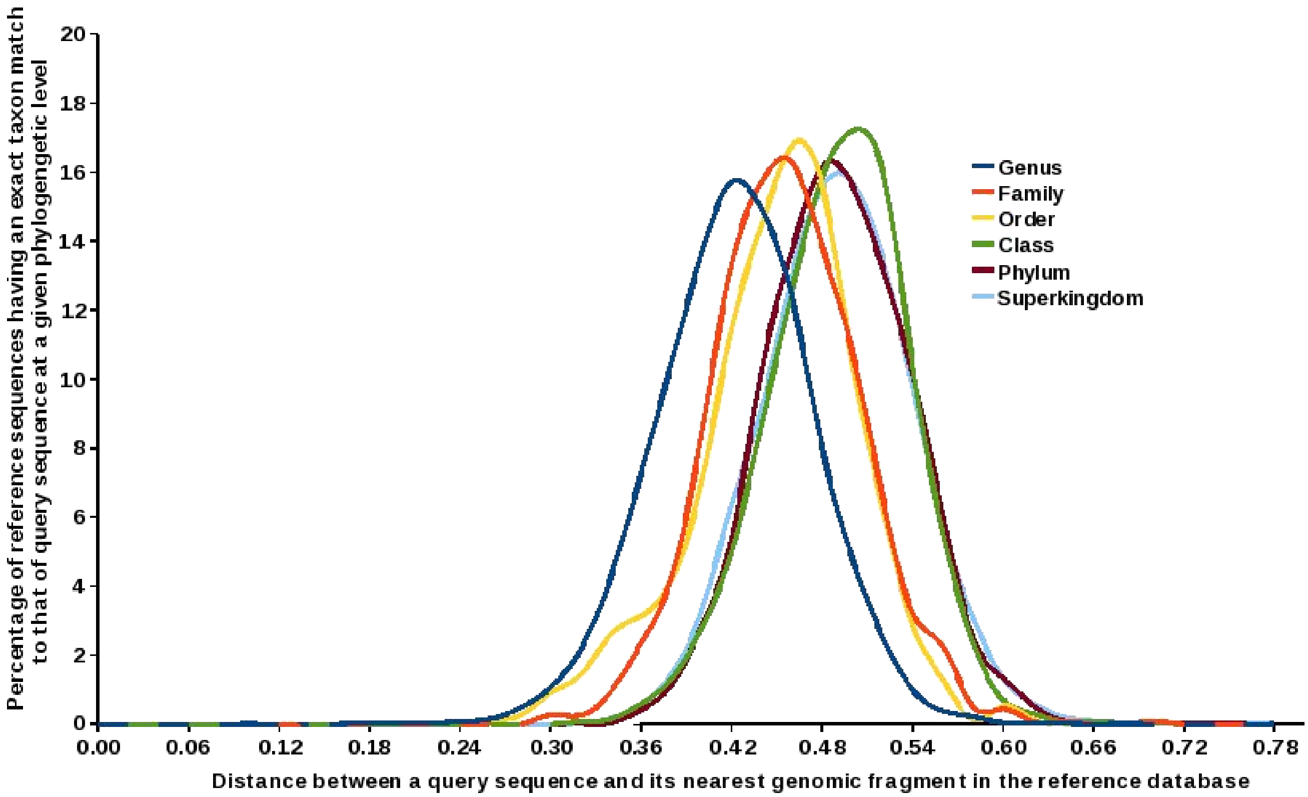

Figure S1C: Plot showing the percentage of sequences in the simulated 454-250 data set in different distance ranges. Sequences tagged as 'diverged from genus', 'diverged from family', 'diverged from order', 'diverged from class', 'diverged from phylum' and 'diverged from superkingdom' are abbreviated as 'Genus', 'Family', 'Order', 'Class', 'Phylum', and 'Super kingdom', respectively.

Results obtained with data set containing sequences of length ~100 bp (454-GS20)

In the absence of a clear distance pattern between sequences tagged to various groups (Figure S1D), assignment of sequences having a distance value < 0.6 was considering from genus levels. Assignment of sequences having the nearest genomic fragment (in reference database) at a distance greater than or equal to 0.6 was considered from class levels.

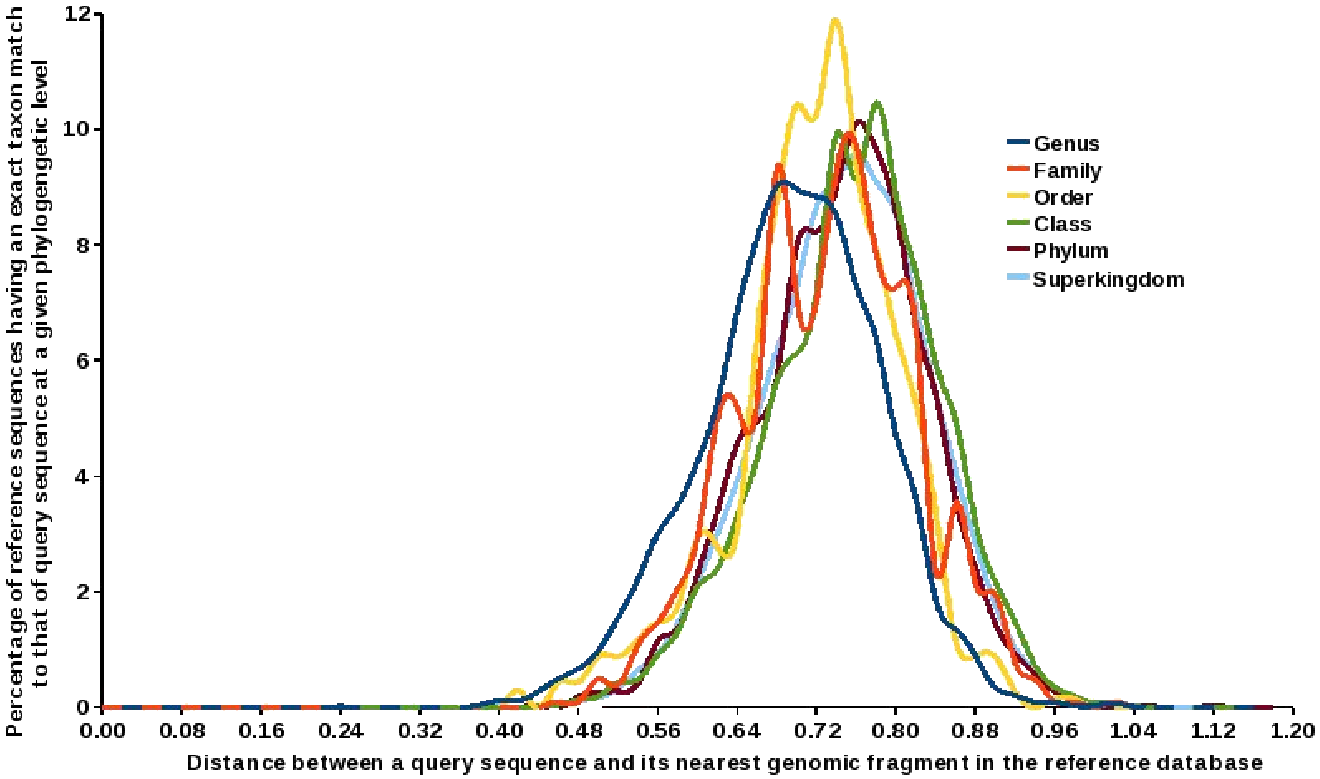

Figure S1D: Plot showing the percentage of sequences in the simulated 454-100 data set in different distance ranges. Sequences tagged as 'diverged from genus', 'diverged from family', 'diverged from order', 'diverged from class', 'diverged from phylum' and 'diverged from super kingdom' are abbreviated as 'Genus', 'Family', 'Order', 'Class', 'Phylum' and 'Super kingdom', respectively.

SUPPLEMENTARY MATERIAL

SECTION B

CHARACTERIZATION OF PARAMETERS FOR NORMALIZATION FUNCTION

The third step in the methodology followed by INDUS involves the following steps. First, the proportions of individual taxa in the identified subset of genome fragments (hereafter referred to as subset proportion/s) are calculated for a given query sequence. Subsequently these proportions are normalized with respect to the relative abundance of that taxon in the reference database (hereafter referred to as database proportion/s). For this purpose, INDUS employs a logarithmic normalization function as represented below.

$$N_i = \frac{P_i\left[a + \log\left(\frac{100}{R_i}\right)\right]}{\sum P_i\left[a + \log\left(\frac{100}{R_i}\right)\right]} * 100$$

Where,

'N_i' represents the normalized percentage of a particular taxon 'i' within the subset of genome fragments identified as closest to the given query sequence,

'P_i' represents the percentage of a particular taxon 'i' within the subset of genome fragments identified as closest to the given query sequence,

'R_i' represents the percentage of a particular taxon 'i' with respect to its representation in the reference database, and

'*a*' represents an integer.

The objective is to characterize the value of *'a'* at different read lengths in order to obtain optimal levels of binning efficiency. For this purpose, four sets of simulated sequences generated from 55 organisms belonging to 55 unique prokaryotic orders (Table S1) were used as training data sets. The 'complete reference database' (consisting of 2.6 million genome fragments from 952 prokaryotic genomes) was modified by complete removal of fragments corresponding to 300 genomes. This modified reference database was used for all the characterization experiments.

The binning efficiency of INDUS at different values of *'a'*, varying from 0 to 10, was determined in terms of a efficiency score which is determined as follows:

$$EfficiencyScore = PC_{Specific} - PC_{Wrong}$$

where, $PC_{Specific}$ refers to percentage change in specific assignments with respect to corresponding data set mean and,
PC_{Wrong} refers to percentage change in wrong assignments with respect to corresponding data set mean.

The efficiency scores obtained at different values of *'a'* at all the four sequence lengths is depicted in Figure S2. For sequence lengths corresponding to Sanger, 454-Titanium and 454-Standard, highest efficiency scores and thereby binning efficiencies were observed at an *'a'* value of about *'2'*. For *'a'* values greater than *'2'*, a progressive decrease in binning efficiency was observed. However at sequence length corresponding to 454-GS20, maximum binning efficiency was observed at an *'a'* value of *'0'* i.e., in the absence of the parameter *'a'*. Therefore in the formula of logarithmic normalization *'a'* represents a positive integer with a value of *'0'* for sequence length corresponding to 454-GS20 and with a value of *'2'* for sequence lengths corresponding to Sanger, 454-Titanium and 454-Standard.

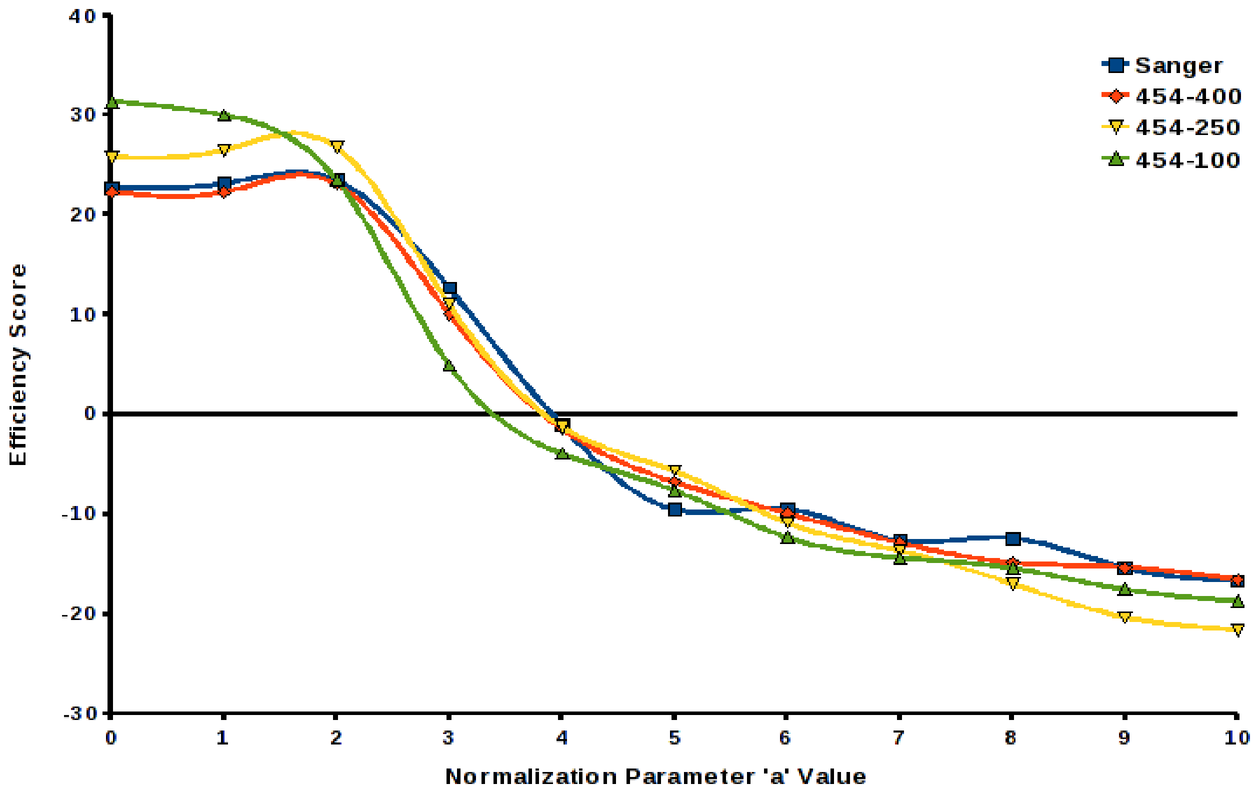

Figure S2: Efficiency scores against various values of the normalization parameter *'a'*

SUPPLEMENTARY MATERIAL

SECTION C

VALIDATION OF THE EFFICIENCY OF NORMALIZATION PROCEDURE

As the existing reference databases are skewed towards organisms of high scientific and/or commercial interest, normalization of subset proportions of individual taxa with respect to their database proportions may reduce the representation bias in the reference database to some extent.

A simple conventional form of normalization where the proportion of each individual taxon in the identified subset is weighted by dividing it with the proportion by which that particular taxon gets represented in the database. As a result the proportions of taxa with higher database representation get down weighted as compared to those of taxa with poor database representation. Such a form of normalization can be represented as follows:

$$N_i = \frac{[\frac{P_i}{R_i}]}{\sum [\frac{P_i}{R_i}]} * 100$$

where,

N_i = Normalized percentage of a particular taxon 'i' within the subset of genome fragments identified as closest to the given query sequence,

P_i = Percentage of a particular taxon 'i' within the subset of genome fragments identified as closest to the given query sequence, and

R_i = Percentage of a particular taxon 'i' with respect to its representation in the reference database

The weighting of subset proportions of taxa with respect to their absolute database proportions may effectively reduce the representation bias in case of poorly represented taxa. However, it may lead to down weighting of subset proportions of highly represented taxa to inappropriately low levels thereby resulting in misclassification of query sequences belonging to those taxa.

In order to overcome the above mentioned limitation associated with the linear normalization, a form of logarithmic normalization, where the subset proportion of a particular taxon is logarithmically weighted with respect to database proportion of that taxon, is considered which can represented as follows:

$$N_i = \frac{P_i[a + \log(\frac{100}{R_i})]}{\sum P_i[a + \log(\frac{100}{R_i})]} * 100$$

where,

'N_i' represents the normalized percentage of a particular taxon 'i' within the subset of genome fragments identified as closest to the given query sequence,

'P_i' represents the percentage of a particular taxon 'i' within the subset of genome fragments identified as closest to the given query sequence,

'R_i' represents the percentage of a particular taxon 'i' with respect to its representation in the reference database, and

'a' represents an integer with a value of either 2 (for query sequences generated using Sanger, 454-Titanium and 454-Standard sequencing technologies) or 0 (for query sequences generated using 454-GS20 sequencing technology).

For the purpose of assessing the impact of normalization procedure in reducing the database representation bias, four validation data sets each comprising of 30000 sequences was constructed from 30 different organisms that can be categorized into two groups with respect their proportion in the reference database. The first group consisted of 14 organisms belonging to sparsely represented taxonomic clades in the reference database and the second group comprised of 16 organisms characterized with highly represented taxonomic clades. The four validation data sets correspond to the sequence lengths of four commonly used sequencing technologies, viz., Sanger (~ 800 bp), 454-Titanium (~ 400 bp), 454-Standard (~ 250 bp) and 454-GS20 (~ 100 bp). The various organisms constituting the data set along with modified

reference database proportions at different taxonomic levels and phylogenetic similarity status with respect to the 55 genomes of the four training data sets are provided in Table S2.

The binning efficiency of INDUS in terms of efficiency score was determined employing no normalization, linear normalization and logarithmic normalization. The results are tabulated in Table S3. The efficiency score was determined as follows:

$$EfficiencyScore = PC_{Specific} - PC_{Wrong}$$

where, $PC_{Specific}$ refers to percentage change in specific assignments with respect to corresponding data set mean and, PC_{Wrong} refers to percentage change in wrong assignments with respect to corresponding data set mean.

The logarithmic normalization exhibited superior binning efficiency compared to linear normalization and no-normalization scenarios. In the absence of normalization, the percentage of specific assignments made by INDUS was slightly higher than that made in the presence of logarithmic normalization and significantly higher than that made in the presence of linear normalization. However, the percentage of wrong assignments in the absence of normalization was considerably higher than that obtained with logarithmic normalization. This resulted in lower efficiency scores for no-normalization compared to logarithmic normalization. The linear normalization underperformed, both in terms of specific and wrong assignments, compared to no-normalization and logarithmic normalization scenarios.

In order to further understand the superior efficiency of logarithmic normalization in reducing the incorrect assignments compared to 'no-normalization' and 'linear normalization', the number of wrong assignments made by INDUS for each of the 30 organisms comprising the validation data sets were individually determined for all the three normalization scenarios. The number of wrong assignments for the 30 organisms of the Sanger data set (for all the three normalization cases) along with their respective modified reference database proportion are tabulated in Table S4A.

For 11 organisms (top/first 11 organisms of Table S4A), logarithmic normalization made lower number of wrong assignments compared to no-normalization. These organisms, except *Shigella dysenteriae Sd197,* had poor percentages of representation in modified reference database at majority of their taxonomic levels. Among organisms with taxonomic clades abundantly represented in the reference database only two organisms, viz., *Bacillus halodurans C-125* and *Pseudomonas mendocina ymp,* recorded higher number of wrong assignments for logarithmic normalization compared to no-normalization. For the remaining organisms both normalization scenarios resulted in no wrong assignments. This may be due to the 'All Known' representation status of these organisms with respect to modified database. Therefore, the relatively less number wrong assignments made against the organisms that are sparsely represented in the database indicate the effectiveness of logarithmic normalization, in reducing the database representation bias, over no-normalization. Only 2 of the 16 highly abundant organisms are associated with higher misclassification numbers for logarithmic normalization compared to no-normalization which further emphasizes its optimal down-weighting of subset proportions for taxa with higher database proportions. Interestingly, for the same exact set of 11 organisms (top/first 11 organisms of Table S4A) the linear normalization achieved lower numbers of wrong assignments compared to logarithmic normalization. However, for 7 out 16 abundantly represented organisms (bottom/last 7 organisms of Table S4A), linear algorithm made wrong assignments at significantly higher numbers (1816 in total) as opposed to 204 wrong assignments made by linear normalization. As a result the overall number of wrong assignments, for 30 organisms, made by linear normalization (4128) is much higher than that of no-normalization (3973) and logarithmic normalization (3489). These numbers indicate that though the linear normalization is able to reduce the database representation bias and thereby reduce the misclassification rates associated with poorly represented taxa, it achieves that at the cost of down-weighting the proportions of abundantly represented taxa to inappropriately low levels. This subsequently resulted in steep rise in the number of overall wrong assignments. In summary, in the case of propagation of representation bias of reference database to the subset of reference genome fragments identified for a given query sequence, the logarithmic normalization is able to reduce such bias thorough optimal down-weighting of subset proportions of taxa with higher database proportions and achieves reasonable levels of assignment accuracy. The similar trend in wrong assignments was observed with lower sequence lengths, i.e., ~ 400 bp (Table S4B), ~ 250 bp (Table S4C) and ~ 100 bp (Table S4D) corresponding to 454-Titanium, 454-Standard and 454-GS20 sequencing technologies respectively.

SUPPLEMENTARY MATERIAL

SECTION D

METHODOLOGY FOR ASSIGNMENT OF TAXA TO QUERY SEQUENCES

The final step in the work-flow followed by INDUS algorithm involves associating a query sequence to a taxon whose (normalized) proportion (within the set of nearest genome fragments) exceeds a predetermined 'threshold' value. This threshold thus is indicative of the phylogenetic uniformity among the set of nearest genome fragments. The objective was to identify an 'optimal' threshold value at which phylogenetic uniformity can be inferred. The emphasis is on the word 'optimal' for the following reason. INDUS iteratively checks at progressively higher taxonomic levels at which the proportion of a taxon (within the set of closest genome fragments) exceeds the predetermined threshold. If this threshold is arbitrarily set high, phylogenetic uniformity would be achieved at relatively high taxonomic levels. This would results in loss of 'assignment specificity'. On the other hand, setting a low threshold would result in loss of 'assignment accuracy', especially in cases of query sequences originating from novel organisms. The experiments described in this document were designed with the objective of identifying a suitable threshold value at which INDUS achieves highest 'assignment efficiency', i.e the right balance between assignment specificity and assignment accuracy.

To determine these threshold values, four training data sets generated from 55 organisms corresponding to 55 unique prokaryotic orders (described in section A of Supplementary Material) were used. The 'complete reference database' (consisting of 2.6 million genome fragments from 952 prokaryotic genomes) was modified by completely removing fragments corresponding to 300 genomes. This modified reference database was used for all experiments.

In order to determine an 'optimal' threshold proportion where INDUS achieves highest binning efficiency, taxonomic assignments for all query sequences (in a given data set) were obtained using all possible threshold values (ranging from 1- 100%). All assignments were categorized into specific and wrong assignments (using the same methodology as explained in section 2.2 of the main manuscript). In order to identify an 'optimal' threshold value, an efficiency score that measures percentage changes in specific and wrong assignments (with respect to the corresponding mean values obtained for the entire data set) was calculated using the following formula.

$$EfficiencyScore = PC_{Specific} - PC_{Wrong}$$

where, $PC_{Specific}$ refers to percentage change in specific assignments with respect to corresponding data set mean and,
PC_{Wrong} refers to percentage change in wrong assignments with respect to corresponding data set mean.

The efficiency score is expected to be high in instances where the percentage decrease in wrong assignments is high with negligible decline in assignment specificity compared to other instances. Results of the above experiments are

depicted in Figure S3. For all four data sets, highest efficiency scores were observed at threshold proportions between 73-79%. The corresponding threshold values for Sanger, 454-400, 454-250 and 454-100 were thus identified as 73%, 76%, 77% and 79% respectively.

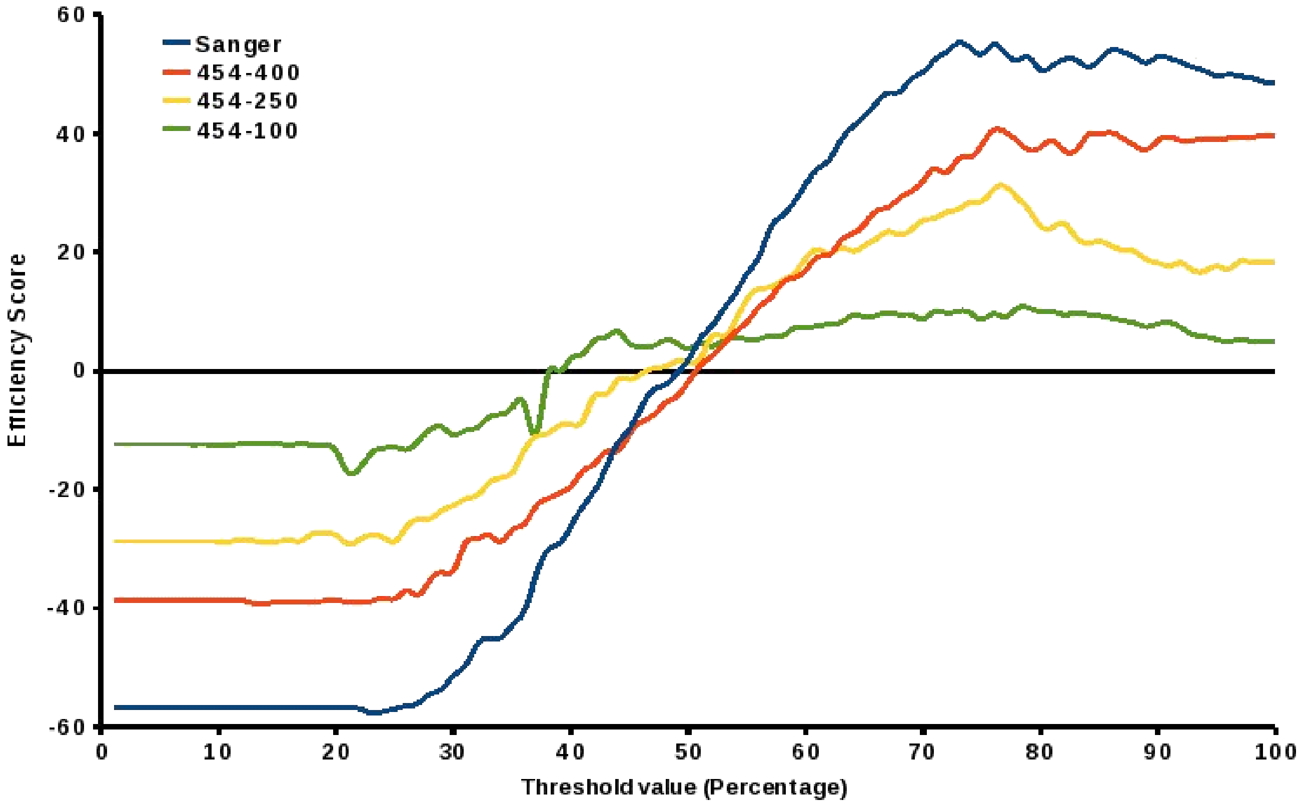

Figure S3: Plot depicting the efficiency scores obtained at various threshold proportions.

Table S1: List of organisms used to generate four different training data sets corresponding to the sequence lengths of Sanger, 454-Titanium, 454-Standard, 454-GS20 sequencing technologies respectively.

S. No.	Organism	Number of Reads
1	*Acidimicrobium ferrooxidans DSM 10331*	1000
2	*Acidithiobacillus ferrooxidans ATCC 23270*	1000
3	*Acidobacterium capsulatum ATCC 51196*	1000
4	*Aeromonas hydrophila subsp. hydrophila ATCC 7966*	1000
5	*Akkermansia muciniphila ATCC BAA-835*	1000
6	*Archaeoglobus fulgidus*	1000
7	*Bacteroides vulgatus ATCC 8482*	1000
8	*Bifidobacterium longum*	1000
9	*Caulobacter sp. K31*	1000
10	*Cenarchaeum symbiosum A*	1000
11	*Coraliomargarita akajimensis*	1000
12	*Deferribacter desulfuricans*	1000
13	*Deinococcus geothermalis DSM 11300*	1000
14	*Dichelobacter nodosus VCS1703A*	1000
15	*Dictyoglomus turgidum DSM 6724*	1000
16	*Fibrobacter succinogenes subsp. succinogenes S85*	1000
17	*Flavobacterium psychrophilum JIP02/86*	1000
18	*Gemmatimonas aurantiaca T-27*	1000
19	*Hahella chejuensis KCTC 2396*	1000
20	*Haloarcula marismortui ATCC 43049*	1000
21	*Halorhodospira halophila SL1*	1000
22	*Halothermothrix orenii H 168*	1000
23	*Herpetosiphon aurantiacus ATCC 23779*	1000
24	*Ignicoccus hospitalis KIN4/I*	1000
25	*Legionella pneumophila str. Corby*	1000
26	*Leptotrichia buccalis DSM 1135*	1000
27	*Methanobrevibacter smithii ATCC 35061*	1000
28	*Methanococcus aeolicus Nankai-3*	1000
29	*Methanoculleus marisnigri JR1*	1000
30	*Methanopyrus kandleri*	1000
31	*Methanosarcina acetivorans*	1000
32	*Methylococcus capsulatus str. Bath*	1000
33	*Natranaerobius thermophilus JW/NM-WN-LF*	1000
34	*Nitrosopumilus maritimus SCM1*	1000
35	*Parvularcula bermudensis HTCC2503*	1000
36	*Pirellula staleyi DSM 6068*	1000
37	*Pyrobaculum arsenaticum DSM 13514*	1000
38	*Rhodobacter sphaeroides ATCC 17025*	1000
39	*Rhodospirillum rubrum ATCC 11170*	1000
40	*Roseiflexus castenholzii DSM 13941*	1000
41	*Rubrobacter xylanophilus DSM 9941*	1000
42	*Salinibacter ruber DSM 13855*	1000
43	*Slackia heliotrinireducens DSM 20476*	1000
44	*Solibacter usitatus Ellin6076*	1000
45	*Sphaerobacter thermophilus DSM 20745*	1000
46	*Sphingomonas wittichii RW1*	1000
47	*Sulfolobus acidocaldarius DSM 639*	1000
48	*Thermoanaerobacter pseudethanolicus ATCC 33223*	1000
49	*Thermococcus onnurineus NA1*	1000
50	*Thermodesulfovibrio yellowstonii DSM 11347*	1000
51	*Thermomicrobium roseum DSM 5159*	1000
52	*Thermoplasma acidophilum*	1000
53	*Thermus thermophilus HB27*	1000
54	*uncultured methanogenic archaeon RC-I*	1000
55	*Vibrio cholerae*	1000
	**Total Number of Reads**	**55000**

Table S2: List of organisms used to validate the effect of normalization on binning efficiency of INDUS. For each individual organism, the number of reads, the representation status* with respect to modified reference database (modified RefDB), the representation percentages of various taxonomic levels in RefDB, the phylogenetic similarity status** with respect to the genomes of training data set are also reported.

S. No	Organism	Reads	Status with respect to modified RefDB*	Percentages of Representation in Modified RefDB at different levels								Status with respect to training data set**
				Strain	Species	Genus	Family	Order	Class	Phylum	Superkingdom	
	**Poorly Represented Organisms:**											
1	*Bacillus halodurans C-125*	1000	Species Unknown	0	0	0.31	1.07	3.37	3.53	4.14	89.72	Phylum Shared
2	*Chlorobium chlorochromatii CaD3*	1000	Genus Unknown	0	0	0	0.15	0.15	0.15	0.15	89.72	Superkingdom Shared
3	*Chloroherpeton thalassium ATCC 35110*	1000	Genus Unknown	0	0	0	0.15	0.15	0.15	0.15	89.72	Superkingdom Shared
4	*Cytophaga hutchinsonii ATCC 33406*	1000	All Known	0.15	0.15	0.15	0.15	0.15	0.15	2.61	89.72	Phylum Shared
5	*Elusimicrobium minutum Pei191*	1000	All Known	0.15	0.15	0.15	0.15	0.15	0.15	0.15	89.72	Superkingdom Shared
6	*Gloeobacter violaceus PCC 7421*	1000	All Known	0.15	0.15	0.15	7.82	0.15	0.15	5.52	89.72	Superkingdom Shared
7	*Lactococcus lactis subsp. lactis Il1403*	1000	Genus Unknown	0	0	0	0.15	0.15	3.53	4.14	89.72	Phylum Shared
8	*Methylacidiphilum infernorum V4*	1000	All Known	0.15	0.15	0.15	0.15	0.15	6.75	0.46	89.72	Phylum Shared
9	*Mycoplasma pneumoniae M129*	1000	Phylum Unknown	0	0	0	0	0	0	0	89.72	Superkingdom Shared
10	*Nostoc sp. PCC 7120*	1000	Class Unknown	0	0	0	0	0	0	5.52	89.72	Superkingdom Shared
11	*Opitutus terrae PB90-1*	1000	All Known	0.15	0.15	0.15	0.15	2.91	0.15	0.46	89.72	Class Shared
12	*Pelodictyon phaeoclathratiforme BU-1*	1000	Genus Unknown	0	0	0	0.15	0.15	0.15	0.15	89.72	Superkingdom Shared
13	*Prosthecochloris aestuarii DSM 271*	1000	Species Unknown	0	0	0.15	0.15	0.15	0.15	0.15	89.72	Superkingdom Shared
14	*Streptococcus pyogenes SSI-1*	1000	Species Unknown	0	0	0.15	0.15	0.15	3.53	4.14	89.72	Phylum Shared
	**Highly Represented Organisms:**											
15	*Bradyrhizobium sp. ORS278*	1000	All Known	0.15	0.15	0.31	0.77	6.6	13.34	57.06	89.72	Class Shared
16	*Candidatus Blochmannia pennsylvanicus str. BPEN*	1000	All Known	0.15	0.15	0.15	13.34	13.34	28.37	57.06	89.72	Class Shared
17	*Clostridium tetani E88*	1000	Species Unknown	0	0	0.46	0.46	0.46	0.46	4.14	89.72	Class Shared
18	*Enterobacter sp. 638*	1000	All Known	0.15	0.15	0.31	13.34	13.34	28.37	57.06	89.72	Class Shared
19	*Escherichia coli ATCC 8739*	1000	All Known	0.15	3.83	3.99	13.34	13.34	28.37	57.06	89.72	Class Shared
20	*Haemophilus influenzae Rd KW20*	1000	Species Unknown	0	0	0.61	1.69	1.69	28.37	57.06	89.72	Class Shared
21	*Klebsiella pneumoniae 342*	1000	All Known	0.15	0.31	0.31	13.34	13.34	28.37	57.06	89.72	Class Shared
22	*Polaromonas sp. JS666*	1000	Species Unknown	0	0	0.15	1.07	7.06	8.9	57.06	89.72	Phylum Shared
23	*Pseudomonas aeruginosa PAO1*	1000	Genus Unknown	0	0	0	0.31	0.92	28.37	57.06	89.72	Class Shared
24	*Pseudomonas mendocina ymp*	1000	Genus Unknown	0	0	0	0.31	0.92	28.37	57.06	89.72	Class Shared
25	*Rhodopseudomonas palustris BisA53*	1000	Genus Unknown	0	0	0	0.77	6.6	13.34	57.06	89.72	Class Shared
26	*Saccharophagus degradans 2-40*	1000	Genus Unknown	0	0	0	0.31	3.83	28.37	57.06	89.72	Class Shared
27	*Salmonella typhimurium LT2*	1000	All Known	0.15	0.15	2.76	13.34	13.34	28.37	57.06	89.72	Class Shared
28	*Shigella dysenteriae Sd197*	1000	All Known	0.15	0.15	1.07	13.34	13.34	28.37	57.06	89.72	Class Shared
29	*Xylella fastidiosa 9a5c*	1000	All Known	0.15	0.61	0.61	0.92	0.92	28.37	57.06	89.72	Class Shared
30	*Yersinia pestis KIM*	1000	All Known	0.15	1.07	1.99	13.34	13.34	28.37	57.06	89.72	Class Shared

*All Known: Genome fragments of that particular microbe is present in the modified RefDB.
*Strain Unknown: Genome fragments of this strain are absent in the modified RefDB.
*Species Unknown: Genome fragments of all strains belonging to this species are absent in the modified RefDB.
*Genus Unknown: Genome fragments of all the strains and species belonging to this genus are absent in modified RefDB.
*Family Unknown: Genome fragments of all genera belonging to this family are absent in the modified RefDB.
*Order Unknown: Genome fragments of all families belonging to this order are absent in the modified RefDB.
*Phylum Unknown: Genome fragments of all classes belonging to this phylum are absent in modified RefDB.

**Class Shared: The training dataset is devoid of genomes that can share either strain or species or genus or family or order with that particular microbe.
**Phylum Shared: The training dataset does not consists of any genomes that belong to either strain or species or genus or family or order or class of that particular microbe.
**Superkingdom Shared: The training dataset does not consists of any genomes that belong to either strain or species or genus or family or order or class of that particular microbe.

Table S3: Validation of binning efficiency of INDUS in the presence and absence of normalization procedure at different read lengths. Results obtained with two different normalization methods, i.e., a linear normali method* and a form of logarithmic normalization method** are compared with those obtained in the absence of any normalization procedure.

Read Length	Type of Normalization	Specific Assignments	Wrong Assignments	Efficiency Score
	**No Normalization**	56.21	13.24	-1.53
**~ 800 bp**	**Linear Normalization**	52.01	13.76	-13.32
	**Logarithmic Normalization**	55.37	10.97	14.90
	**No Normalization**	43.21	14.69	1.70
**~ 400 bp**	**Linear Normalization**	38.19	15.49	-16.16
	**Logarithmic Normalization**	41.80	12.40	14.44
	**No Normalization**	34.82	16.76	8.25
**~ 250 bp**	**Linear Normalization**	26.52	18.55	-28.93
	**Logarithmic Normalization**	33.52	14.05	20.59
	**No Normalization**	20.75	15.55	14.88
**~ 100 bp**	**Linear Normalization**	12.60	16.64	-38.83
	**Logarithmic Normalization**	19.27	12.89	24.06

Table S4A: Number of wrong assignments obtained for each of 30 organisms (14 poorly represented + 16 highly represented) at Sanger sequence lengths (~ 800 bp) during validation of INDUS normalization emp logarithmic normalization, no normalization and linear normalization procedures respectively. The percentages of representation of each organism, at different taxonomic levels, in the modified reference database (mo RefDB) are also presented.

SANGER															
**S. No.**	**Organisms**	**Status***	**Reads**	**Wrong Assignments**			**Reduction in Wrong Assignments of Logarithmic Normalization with respect to**		**Percentages of Representation in Modified RefDB at different levels**						
				Logarithmic Normalization	No Normalization	Linear Normalization	No Normalization	Linear Normalization	Species	Genus	Family	Order	Class	Phylum	Superkingdom
1	Prosthecochloris aestuarii DSM 271	Species Unknown	1000	235	347	166	112	-69	0	0.15	0.15	0.15	0.15	0.15	89.72
2	Pelodictyon phaeoclathratiforme BU-1	Genus Unknown	1000	247	346	158	99	-89	0	0	0.15	0.15	0.15	0.15	89.72
3	Nostoc sp. PCC 7120	Class Unknown	1000	312	384	100	72	-212	0	0	0	0	0	5.52	89.72
4	Streptococcus pyogenes SSI-1	Species Unknown	1000	298	356	277	58	-21	0	0.15	0.15	0.15	3.53	4.14	89.72
5	Gloeobacter violaceus PCC 7421	All Known	1000	0	57	50	57	50	0.15	0.15	7.82	0.15	0.15	5.52	89.72
6	Shigella dysenteriae Sd197	All Known	1000	333	386	271	53	-62	0.15	1.07	13.34	13.34	28.37	57.06	89.72
7	Opitutus terrae PB90-1	All Known	1000	62	106	0	44	-62	0.15	0.15	0.15	2.91	0.15	0.46	89.72
8	Chloroherpeton thalassium ATCC 35110	Genus Unknown	1000	485	527	377	42	-108	0	0	0.15	0.15	0.15	0.15	89.72
9	Chlorobium chlorochromatii CaD3	Genus Unknown	1000	720	747	504	27	-216	0	0	0.15	0.15	0.15	0.15	89.72
10	Lactococcus lactis subsp. lactis Il1403	Genus Unknown	1000	110	133	45	23	-65	0	0	0.15	0.15	3.53	4.14	89.72
11	Mycoplasma pneumoniae M129	Phylum Unknown	1000	483	500	364	17	-119	0	0	0	0	0	0	89.72
12	Bradyrhizobium sp. ORS278	All Known	1000	0	0	0	0	0	0.15	0.31	0.77	6.6	13.34	57.06	89.72
13	Enterobacter sp. 638	All Known	1000	0	0	0	0	0	0.15	0.31	13.34	13.34	28.37	57.06	89.72
14	Elusimicrobium minutum Pei191	All Known	1000	0	0	0	0	0	0.15	0.15	0.15	0.15	0.15	0.15	89.72
15	Cytophaga hutchinsonii ATCC 33406	All Known	1000	0	0	0	0	0	0.15	0.15	0.15	0.15	0.15	2.61	89.72
16	Methylacidiphilum infernorum V4	All Known	1000	0	0	0	0	0	0.15	0.15	0.15	0.15	6.75	0.46	89.72
17	Klebsiella pneumoniae 342	All Known	1000	0	0	0	0	0	0.31	0.31	13.34	13.34	28.37	57.06	89.72
18	Haemophilus influenzae Rd KW20	Species Unknown	1000	0	0	0	0	0	0	0.61	1.69	1.69	28.37	57.06	89.72
19	Saccharophagus degradans 2-40	Genus Unknown	1000	0	0	0	0	0	0	0	0.31	3.83	28.37	57.06	89.72
20	Yersinia pestis KIM	All Known	1000	0	0	0	0	0	1.07	1.99	13.34	13.34	28.37	57.06	89.72
21	Xylella fastidiosa 9a5c	All Known	1000	0	0	0	0	0	0.61	0.61	0.92	0.92	28.37	57.06	89.72
22	Candidatus Blochmannia pennsylvanicus str. BPEN	All Known	1000	0	0	0	0	0	0.15	0.15	13.34	13.34	28.37	57.06	89.72
23	Salmonella typhimurium LT2	All Known	1000	0	0	0	0	0	0.15	2.76	13.34	13.34	28.37	57.06	89.72
24	Bacillus halodurans C-125	Species Unknown	1000	100	84	133	-16	33	0	0.31	1.07	3.37	3.53	4.14	89.72
25	Escherichia coli ATCC 8739	All Known	1000	0	0	173	0	173	3.83	3.99	13.34	13.34	28.37	57.06	89.72
26	Polaromonas sp. JS666	Species Unknown	1000	0	0	248	0	248	0	0.15	1.07	7.06	8.9	57.06	89.72
27	Pseudomonas mendocina ymp	Genus Unknown	1000	104	0	382	-104	278	0	0	0.31	0.92	28.37	57.06	89.72
28	Clostridium tetani E88	Species Unknown	1000	0	0	284	0	284	0	0.46	0.46	0.46	0.46	4.14	89.72
29	Rhodopseudomonas palustris BisA53	Genus Unknown	1000	0	0	296	0	296	0	0	0.77	6.6	13.34	57.06	89.72
30	Pseudomonas aeruginosa PAO1	Genus Unknown	1000	0	0	300	0	300	0	0	0.31	0.92	28.37	57.06	89.72
	Total Number of Wrong Assignments		30000	3489	3973	4128									

*All Known: Genome fragments of that particular microbe is present in the modified RefDB.
*Strain Unknown: Genome fragments of this strain are absent in the modified RefDB.
*Species Unknown: Genome fragments of all strains belonging to this species are absent in the modified RefDB.
*Genus Unknown: Genome fragments of all the strains and species belonging to this genus are absent in modified RefDB.
*Family Unknown: Genome fragments of all genera belonging to this family are absent in the modified RefDB.
*Order Unknown: Genome fragments of all families belonging to this order are absent in the modified RefDB.
*Phylum Unknown: Genome fragments of all classes belonging to this phylum are absent in modified RefDB.

Table S4B: Number of wrong assignments obtained for each of 30 organisms (14 poorly represented + 16 highly represented) at 454-Titanium sequence lengths (~ 400 bp) during validation of INDUS normalization employing logarithmic normalization, no normalization and linear normalization procedures respectively. The percentages of representation of each organism, at different taxonomic levels, in the modified reference database (modified RefDB) are also presented.

454400																
S. No.	Organisms	Status*	Reads	Wrong Assignments			Reduction in Wrong Assignments of Logarithmic Normalization with respect to		Percentages of Representation in Modified RefDB at different levels							
				Logarithmic Normalization	No Normalization	Linear Normalization	No Normalization	Linear Normalization	Species	Genus	Family	Order	Class	Phylum	Superkingdom
1	Opitutus terrae PB90-1	All Known	1000	310	399	154	89	-156	0.15	0.15	0.15	2.91	0.15	0.46	89.72
2	Streptococcus pyogenes SSI-1	Species Unknown	1000	260	336	236	76	-24	0	0.15	0.15	0.15	3.53	4.14	89.72
3	Prosthecochloris aestuarii DSM 271	Species Unknown	1000	214	285	235	71	21	0	0.15	0.15	0.15	0.15	0.15	89.72
4	Pelodictyon phaeoclathratiforme BU-1	Genus Unknown	1000	222	293	159	71	-63	0	0	0.15	0.15	0.15	0.15	89.72
5	Bacillus halodurans C-125	Species Unknown	1000	100	163	185	63	85	0	0.31	1.07	3.37	3.53	4.14	89.72
6	Nostoc sp. PCC 7120	Class Unknown	1000	278	330	92	52	-186	0	0	0	0	0	5.52	89.72
7	Mycoplasma pneumoniae M129	Phylum Unknown	1000	368	416	262	48	-106	0	0	0	0	0	0	89.72
8	Chloroherpeton thalassium ATCC 35110	Genus Unknown	1000	436	483	324	47	-112	0	0	0.15	0.15	0.15	0.15	89.72
9	Elusimicrobium minutum Pei191	All Known	1000	0	42	0	42	0	0.15	0.15	0.15	0.15	0.15	0.15	89.72
10	Chlorobium chlorochromatii CaD3	Genus Unknown	1000	571	612	341	41	-230	0	0	0.15	0.15	0.15	0.15	89.72
11	Lactococcus lactis subsp. lactis Il1403	Genus Unknown	1000	90	110	128	20	38	0	0	0.15	0.15	3.53	4.14	89.72
12	Cytophaga hutchinsonii ATCC 33406	All Known	1000	79	90	0	11	-79	0.15	0.15	0.15	0.15	0.15	2.61	89.72
13	Gloeobacter violaceus PCC 7421	All Known	1000	207	202	133	-5	-74	0.15	0.15	7.82	0.15	0.15	5.52	89.72
14	Saccharophagus degradans 2-40	Genus Unknown	1000	0	0	0	0	0	0	0	0.31	3.83	28.37	57.06	89.72
15	Escherichia coli ATCC 8739	All Known	1000	0	0	0	0	0	3.83	3.99	13.34	13.34	28.37	57.06	89.72
16	Methylacidiphilum infernorum V4	All Known	1000	0	0	0	0	0	0.15	0.15	0.15	0.15	6.75	0.46	89.72
17	Shigella dysenteriae Sd197	All Known	1000	0	0	0	0	0	0.15	1.07	13.34	13.34	28.37	57.06	89.72
18	Salmonella typhimurium LT2	All Known	1000	0	0	0	0	0	0.15	2.76	13.34	13.34	28.37	57.06	89.72
19	Yersinia pestis KIM	All Known	1000	0	0	21	0	21	1.07	1.99	13.34	13.34	28.37	57.06	89.72
20	Candidatus Blochmannia pennsylvanicus str. BPEN	All Known	1000	0	0	42	0	42	0.15	0.15	13.34	13.34	28.37	57.06	89.72
21	Enterobacter sp. 638	All Known	1000	0	0	50	0	50	0.15	0.31	13.34	13.34	28.37	57.06	89.72
22	Xylella fastidiosa 9a5c	All Known	1000	0	0	60	0	60	0.61	0.61	0.92	0.92	28.37	57.06	89.72
23	Haemophilus influenzae Rd KW20	Species Unknown	1000	47	44	118	-3	71	0	0.61	1.69	1.69	28.37	57.06	89.72
24	Klebsiella pneumoniae 342	All Known	1000	0	0	96	0	96	0.31	0.31	13.34	13.34	28.37	57.06	89.72
25	Rhodopseudomonas palustris BisA53	Genus Unknown	1000	201	192	356	-9	155	0	0	0.77	6.6	13.34	57.06	89.72
26	Bradyrhizobium sp. ORS278	All Known	1000	76	68	253	-8	177	0.15	0.31	0.77	6.6	13.34	57.06	89.72
27	Pseudomonas mendocina ymp	Genus Unknown	1000	168	144	357	-24	189	0	0	0.31	0.92	28.37	57.06	89.72
28	Pseudomonas aeruginosa PAO1	Genus Unknown	1000	181	152	429	-29	248	0	0	0.31	0.92	28.37	57.06	89.72
29	Polaromonas sp. JS666	Species Unknown	1000	52	46	332	-6	280	0	0.15	1.07	7.06	8.9	57.06	89.72
30	Clostridium tetani E88	Species Unknown	1000	0	0	284	0	284	0	0.46	0.46	0.46	0.46	4.14	89.72
	Total Number of Wrong Assignments		30000	3860	4407	4647									

*All Known: Genome fragments of that particular microbe is present in the modified RefDB.
*Strain Unknown: Genome fragments of this strain are absent in the modified RefDB.
*Species Unknown: Genome fragments of all strains belonging to this species are absent in the modified RefDB.
*Genus Unknown: Genome fragments of all the strains and species belonging to this genus are absent in modified RefDB.
*Family Unknown: Genome fragments of all genera belonging to this family are absent in the modified RefDB.
*Order Unknown: Genome fragments of all families belonging to this order are absent in the modified RefDB.
*Phylum Unknown: Genome fragments of all classes belonging to this phylum are absent in modified RefDB.

Table S4C: Number of wrong assignments obtained for each of 30 organisms (14 poorly represented + 16 highly represented) at 454-Standard sequence lengths (~ 250 bp) during validation of INDUS normalization employing logarithmic normalization, no normalization and linear normalization procedures respectively. The percentages of representation of each organism, at different taxonomic levels, in the modified reference database (modified RefDB) are also presented.

454250															
**S. No.**	**Organisms**	**Status***	**Reads**	**Wrong Assignments**			**Reduction in Wrong Assignments of Logarithmic Normalization with respect to**		**Percentages of Representation in Modified RefDB at different levels**						
				Logarithmic Normalization	No Normalization	Linear Normalization	No Normalization	Linear Normalization	Species	Genus	Family	Order	Class	Phylum	Superkingdom
1	Pelodictyon phaeoclathratiforme BU-1	Genus Unknown	1000	181	307	222	126	41	0	0	0.15	0.15	0.15	0.15	89.72
2	Nostoc sp. PCC 7120	Class Unknown	1000	220	319	102	99	-118	0	0	0	0	0	5.52	89.72
3	Streptococcus pyogenes SSI-1	Species Unknown	1000	242	341	170	99	-72	0	0.15	0.15	0.15	3.53	4.14	89.72
4	Opitutus terrae PB90-1	All Known	1000	442	540	261	98	-181	0.15	0.15	0.15	2.91	0.15	0.46	89.72
5	Pseudomonas mendocina ymp	Genus Unknown	1000	157	236	299	79	142	0	0	0.31	0.92	28.37	57.06	89.72
6	Gloeobacter violaceus PCC 7421	All Known	1000	282	360	204	78	-78	0.15	0.15	7.82	0.15	0.15	5.52	89.72
7	Bacillus halodurans C-125	Species Unknown	1000	117	189	220	72	103	0	0.31	1.07	3.37	3.53	4.14	89.72
8	Chloroherpeton thalassium ATCC 35110	Genus Unknown	1000	375	444	329	69	-46	0	0	0.15	0.15	0.15	0.15	89.72
9	Chlorobium chlorochromatii CaD3	Genus Unknown	1000	531	597	381	66	-150	0	0	0.15	0.15	0.15	0.15	89.72
10	Cytophaga hutchinsonii ATCC 33406	All Known	1000	94	154	0	60	-94	0.15	0.15	0.15	0.15	0.15	2.61	89.72
11	Prosthecochloris aestuarii DSM 271	Species Unknown	1000	216	274	265	58	49	0	0.15	0.15	0.15	0.15	0.15	89.72
12	Methylacidiphilum infernorum V4	All Known	1000	0	47	0	47	0	0.15	0.15	0.15	0.15	6.75	0.46	89.72
13	Elusimicrobium minutum Pei191	All Known	1000	81	110	62	29	-19	0.15	0.15	0.15	0.15	0.15	0.15	89.72
14	Lactococcus lactis subsp. lactis Il1403	Genus Unknown	1000	81	105	171	24	90	0	0	0.15	0.15	3.53	4.14	89.72
15	Mycoplasma pneumoniae M129	Phylum Unknown	1000	350	371	264	21	-86	0	0	0	0	0	0	89.72
16	Saccharophagus degradans 2-40	Genus Unknown	1000	0	0	34	0	34	0	0	0.31	3.83	28.37	57.06	89.72
17	Salmonella typhimurium LT2	All Known	1000	0	0	41	0	41	0.15	2.76	13.34	13.34	28.37	57.06	89.72
18	Shigella dysenteriae Sd197	All Known	1000	0	0	47	0	47	0.15	1.07	13.34	13.34	28.37	57.06	89.72
19	Escherichia coli ATCC 8739	All Known	1000	0	0	49	0	49	3.83	3.99	13.34	13.34	28.37	57.06	89.72
20	Rhodopseudomonas palustris BisA53	Genus Unknown	1000	220	205	287	-15	67	0	0	0.77	6.6	13.34	57.06	89.72
21	Bradyrhizobium sp. ORS278	All Known	1000	194	169	283	-25	89	0.15	0.31	0.77	6.6	13.34	57.06	89.72
22	Haemophilus influenzae Rd KW20	Species Unknown	1000	0	0	100	0	100	0	0.61	1.69	1.69	28.37	57.06	89.72
23	Enterobacter sp. 638	All Known	1000	0	0	101	0	101	0.15	0.31	13.34	13.34	28.37	57.06	89.72
24	Yersinia pestis KIM	All Known	1000	49	49	156	0	107	1.07	1.99	13.34	13.34	28.37	57.06	89.72
25	Candidatus Blochmannia pennsylvanicus str. BPEN	All Known	1000	0	0	108	0	108	0.15	0.15	13.34	13.34	28.37	57.06	89.72
26	Klebsiella pneumoniae 342	All Known	1000	0	0	158	0	158	0.31	0.31	13.34	13.34	28.37	57.06	89.72
27	Xylella fastidiosa 9a5c	All Known	1000	0	0	170	0	170	0.61	0.61	0.92	0.92	28.37	57.06	89.72
28	Pseudomonas aeruginosa PAO1	Genus Unknown	1000	179	94	373	-85	194	0	0	0.31	0.92	28.37	57.06	89.72
29	Clostridium tetani E88	Species Unknown	1000	43	56	330	13	287	0	0.46	0.46	0.46	0.46	4.14	89.72
30	Polaromonas sp. JS666	Species Unknown	1000	69	60	379	-9	310	0	0.15	1.07	7.06	8.9	57.06	89.72
	Total Number of Wrong Assignments		30000	4123	5027	5566									

*All Known: Genome fragments of that particular microbe is present in the modified RefDB.
*Strain Unknown: Genome fragments of this strain are absent in the modified RefDB.
*Species Unknown: Genome fragments of all strains belonging to this species are absent in the modified RefDB.
*Genus Unknown: Genome fragments of all the strains and species belonging to this genus are absent in modified RefDB.
*Family Unknown: Genome fragments of all genera belonging to this family are absent in the modified RefDB.
*Order Unknown: Genome fragments of all families belonging to this order are absent in the modified RefDB.
*Phylum Unknown: Genome fragments of all classes belonging to this phylum are absent in modified RefDB.

Table S4D: Number of wrong assignments obtained for each of 30 organisms (14 poorly represented + 16 highly represented) at 454-GS20 sequence lengths (~ 100 bp) during validation of INDUS normalization employing logarithmic normalization, no normalization and linear normalization procedures respectively. The percentages of representation of each organism, at different taxonomic levels, in the modified reference database (modified RefDB) are also presented.

454100															
**S. No.**	**Organisms**	**Status***	**Reads**	**Wrong Assignments**			**Reduction in Wrong Assignments of Logarithmic Normalization with respect to**		**Percentages of Representation in Modified RefDB at different levels**						
				Logarithmic Normalization	No Normalization	Linear Normalization	No Normalization	Linear Normalization	Species	Genus	Family	Order	Class	Phylum	Superkingdom
1	Chlorobium chlorochromatii CaD3	Genus Unknown	1000	277	400	202	123	-75	0	0	0.15	0.15	0.15	0.15	89.72
2	Cytophaga hutchinsonii ATCC 33406	All Known	1000	86	196	53	110	-33	0.15	0.15	0.15	0.15	0.15	2.61	89.72
3	Elusimicrobium minutum Pei191	All Known	1000	87	191	73	104	-14	0.15	0.15	0.15	0.15	0.15	0.15	89.72
4	Pelodictyon phaeoclathratiforme BU-1	Genus Unknown	1000	160	256	191	96	31	0	0	0.15	0.15	0.15	0.15	89.72
5	Nostoc sp. PCC 7120	Class Unknown	1000	117	213	44	96	-73	0	0	0	0	0	5.52	89.72
6	Gloeobacter violaceus PCC 7421	All Known	1000	372	464	368	92	-4	0.15	0.15	7.82	0.15	0.15	5.52	89.72
7	Streptococcus pyogenes SSI-1	Species Unknown	1000	91	181	98	90	7	0	0.15	0.15	0.15	3.53	4.14	89.72
8	Methylacidiphilum infernorum V4	All Known	1000	0	90	23	90	23	0.15	0.15	0.15	0.15	6.75	0.46	89.72
9	Prosthecochloris aestuarii DSM 271	Species Unknown	1000	135	218	181	83	46	0	0.15	0.15	0.15	0.15	0.15	89.72
10	Chloroherpeton thalassium ATCC 35110	Genus Unknown	1000	252	331	248	79	-4	0	0	0.15	0.15	0.15	0.15	89.72
11	Opitutus terrae PB90-1	All Known	1000	522	599	487	77	-35	0.15	0.15	0.15	2.91	0.15	0.46	89.72
12	Mycoplasma pneumoniae M129	Phylum Unknown	1000	267	336	245	69	-22	0	0	0	0	0	0	89.72
13	Bradyrhizobium sp. ORS278	All Known	1000	219	210	196	-9	-23	0.15	0.31	0.77	6.6	13.34	57.06	89.72
14	Pseudomonas mendocina ymp	Genus Unknown	1000	236	233	220	-3	-16	0	0	0.31	0.92	28.37	57.06	89.72
15	Klebsiella pneumoniae 342	All Known	1000	56	54	46	-2	-10	0.31	0.31	13.34	13.34	28.37	57.06	89.72
16	Lactococcus lactis subsp. lactis Il1403	Genus Unknown	1000	73	126	73	53	0	0	0	0.15	0.15	3.53	4.14	89.72
17	Rhodopseudomonas palustris BisA53	Genus Unknown	1000	195	184	214	-11	19	0	0	0.77	6.6	13.34	57.06	89.72
18	Haemophilus influenzae Rd KW20	Species Unknown	1000	0	0	24	0	24	0	0.61	1.69	1.69	28.37	57.06	89.72
19	Salmonella typhimurium LT2	All Known	1000	0	0	43	0	43	0.15	2.76	13.34	13.34	28.37	57.06	89.72
20	Bacillus halodurans C-125	Species Unknown	1000	108	157	181	49	73	0	0.31	1.07	3.37	3.53	4.14	89.72
21	Saccharophagus degradans 2-40	Genus Unknown	1000	0	0	97	0	97	0	0	0.31	3.83	28.37	57.06	89.72
22	Pseudomonas aeruginosa PAO1	Genus Unknown	1000	186	95	293	-91	107	0	0	0.31	0.92	28.37	57.06	89.72
23	Yersinia pestis KIM	All Known	1000	0	0	112	0	112	1.07	1.99	13.34	13.34	28.37	57.06	89.72
24	Shigella dysenteriae Sd197	All Known	1000	0	0	113	0	113	0.15	1.07	13.34	13.34	28.37	57.06	89.72
25	Enterobacter sp. 638	All Known	1000	0	0	130	0	130	0.15	0.31	13.34	13.34	28.37	57.06	89.72
26	Polaromonas sp. JS666	Species Unknown	1000	81	71	221	-10	140	0	0.15	1.07	7.06	8.9	57.06	89.72
27	Escherichia coli ATCC 8739	All Known	1000	0	0	142	0	142	3.83	3.99	13.34	13.34	28.37	57.06	89.72
28	Xylella fastidiosa 9a5c	All Known	1000	0	0	144	0	144	0.61	0.61	0.92	0.92	28.37	57.06	89.72
29	Candidatus Blochmannia pennsylvanicus str. BPEN	All Known	1000	0	0	201	0	201	0.15	0.15	13.34	13.34	28.37	57.06	89.72
30	Clostridium tetani E88	Species Unknown	1000	0	59	328	59	328	0	0.46	0.46	0.46	0.46	4.14	89.72
	Total Number of Wrong Assignments		30000	3520	4664	4991									

*All Known: Genome fragments of that particular microbe is present in the modified RefDB.
*Strain Unknown: Genome fragments of this strain are absent in the modified RefDB.
*Species Unknown: Genome fragments of all strains belonging to this species are absent in the modified RefDB.
*Genus Unknown: Genome fragments of all the strains and species belonging to this genus are absent in modified RefDB.
*Family Unknown: Genome fragments of all genera belonging to this family are absent in the modified RefDB.
*Order Unknown: Genome fragments of all families belonging to this order are absent in the modified RefDB.
*Phylum Unknown: Genome fragments of all classes belonging to this phylum are absent in modified RefDB.
