# Supplementary material for: INDUS - a composition-based approach for rapid and accurate taxonomic classification of metagenomic sequences
Source: BMC Genomics. 2011 Nov 30;12(Suppl 3):S4. doi: 10.1186/1471-2164-12-S3-S4 (PMC3333187; doi:10.1186/1471-2164-12-S3-S4)
Supplement: Additional file 2 — List of organisms constituting the four test data sets A document containing the list constituting the four simulated test data sets and their status with respect to the modified reference database. [file 1471-2164-12-S3-S4-S2.pdf]

Supplementary Table 1: List of organisms used for constructing simulated test data sets. For each test organism, the representation status* with respect to modified reference database (modified RefDB) and the phylogenetic similarity status** with respect to the genomes of training data set are also presented.

**Sanger data set**				
**S. No.**	**Organism**	**Number of sequences**	**Status with respect to modified RefDB***	**Status with respect to training data set****
1.	*Acaryochloris marina MBIC11017*	1000	All Known	Superkingdom Shared
2.	*Agrobacterium tumefaciens str. C58*	1000	Species Unknown	Class Shared
3.	*Aquifex aeolicus VF5*	1000	All Known	Superkingdom Shared
4	*Bacillus halodurans C-125*	1000	Species Unknown	Phylum Shared
5.	*Bdellovibrio bacteriovorus HD100*	1000	Order Unknown	Phylum Shared
6.	*Bordetella pertussis Tohama I*	1000	Family Unknown	Phylum Shared
7.	*Bradyrhizobium sp. BTAi1*	1000	Species Unknown	Class Shared
8.	*Burkholderia cenocepacia AU 1054*	1000	All Known	Phylum Shared
9.	*Campylobacter jejuni subsp. jejuni NCTC 11168*	1000	Genus Unknown	Phylum Shared
10.	*Chlamydophila pneumoniae AR39*	1000	Genus Unknown	Superkingdom Shared
11.	*Chlorobium chlorochromatii CaD3*	1000	Genus Unknown	Superkingdom Shared
12.	*Clostridium tetani E88*	1000	Species Unknown	Class Shared
13.	*Corynebacterium diphtheriae NCTC 13129*	1000	Species Unknown	Class Shared
14.	*Francisella philomiragia subsp. philomiragia ATCC 25017*	1000	Species Unknown	Class Shared
15.	*Haemophilus influenzae Rd KW20*	1000	Species Unknown	Class Shared
16.	*Helicobacter pylori J99*	1000	Strain Unknown	Phylum Shared
17.	*Lactobacillus acidophilus NCFM*	1000	Family Unknown	Phylum Shared
18.	*Leptospira interrogans serovar Lai str. 56601*	1000	Family Unknown	Superkingdom Shared
19.	*Mycoplasma pneumoniae M129*	1000	Phylum Unknown	Superkingdom Shared
20.	*Neisseria meningitidis MC58*	1000	Genus Unknown	Phylum Shared
21.	*Nostoc sp. PCC 7120*	1000	Order Unknown	Superkingdom Shared
22.	*Polaromonas sp. JS666*	1000	Species Unknown	Phylum Shared
23.	*Pseudomonas aeruginosa PAO1*	1000	Genus Unknown	Class Shared
24.	*Pseudomonas mendocina ymp*	1000	Genus Unknown	Class Shared
25.	*Rhizobium etli CFN 42*	1000	Species Unknown	Class Shared
26.	*Rhodopseudomonas palustris BisA53*	1000	Genus Unknown	Class Shared
27.	*Rickettsia bellii RML369-C*	1000	Genus Unknown	Class Shared
28.	*Saccharophagus degradans 2-40*	1000	Genus Unknown	Class Shared
29.	*Shewanella baltica OS185*	1000	Species Unknown	Class Shared
30.	*Staphylococcus aureus subsp. aureus*	1000	Species Unknown	Phylum Shared
31.	*Streptomyces coelicolor A3(2)*	1000	Species Unknown	Class Shared
32.	*Thermotoga maritima MSB8*	1000	Genus Unknown	Superkingdom Shared
33.	*Trichodesmium erythraeum IMS101*	1000	Order Unknown	Superkingdom Shared
34.	*Wigglesworthia glossinidia endosymbiont of Glossina brevipalpis*	1000	Genus Unknown	Class Shared
35.	*Xanthomonas campestris pv. campestris str. ATCC 33913*	1000	Genus Unknown	Class Shared

*All Known: Genome fragments of that particular microbe is present in the modified RefDB.
*Strain Unknown: Genome fragments of this strain are absent in the modified RefDB.
*Species Unknown: Genome fragments of all strains belonging to this species are absent in the modified RefDB.
*Genus Unknown: Genome fragments of all the strains and species belonging to this genus are absent in modified RefDB.
*Family Unknown: Genome fragments of all genera belonging to this family are absent in the modified RefDB.
*Order Unknown: Genome fragments of all families belonging to this order are absent in the modified RefDB.
*Phylum Unknown: Genome fragments of all classes belonging to this phylum are absent in modified RefDB.

**Class Shared: The training data set is devoid of genomes that can share either strain or species or genus or family or order with that particular microbe.
**Phylum Shared: The training data set does not consists of any genomes that belong to either strain or species or genus or family or order or class of that particular microbe.
**Superkingdom Shared: The training data set does not consists of any genomes that belong to either strain or species or genus or family or order or class or phylum of that particular microbe.

Supplementary Table 1: (contd)

**454-400 and 454-250 data sets**				
**S. No.**	**Organism**	**Number of sequences**	**Status with respect to modified RefDB***	**Status with respect to training data set****
1.	*Acidovorax citrulli AAC00-1*	1000	All Known	Phylum Shared
2.	*Agrobacterium tumefaciens str. C58*	1000	Species Unknown	Class Shared
3.	*Aquifex aeolicus VF5*	1000	All Known	Superkingdom Shared
4	*Bacillus halodurans C-125*	1000	Species Unknown	Phylum Shared
5.	*Bdellovibrio bacteriovorus HD100*	1000	Order Unknown	Phylum Shared
6.	*Bordetella pertussis Tohama I*	1000	Family Unknown	Phylum Shared
7.	*Bradyrhizobium sp. BTAi1*	1000	Species Unknown	Class Shared
8.	*Burkholderia cenocepacia AU 1054*	1000	All Known	Phylum Shared
9.	*Campylobacter jejuni subsp. jejuni NCTC 11168*	1000	Genus Unknown	Phylum Shared
10.	*Chlamydophila pneumoniae AR39*	1000	Genus Unknown	Superkingdom Shared
11.	*Chlorobium chlorochromatii CaD3*	1000	Genus Unknown	Superkingdom Shared
12.	*Clostridium tetani E88*	1000	Species Unknown	Class Shared
13.	*Corynebacterium diphtheriae NCTC 13129*	1000	Species Unknown	Class Shared
14.	*Francisella philomiragia subsp. philomiragia ATCC 25017*	1000	Species Unknown	Class Shared
15.	*Haemophilus influenzae Rd KW20*	1000	Species Unknown	Class Shared
16.	*Helicobacter pylori J99*	1000	Strain Unknown	Phylum Shared
17.	*Lactobacillus acidophilus NCFM*	1000	Family Unknown	Phylum Shared
18.	*Leptospira interrogans serovar Lai str. 56601*	1000	Family Unknown	Superkingdom Shared
19.	*Mycoplasma pneumoniae M129*	1000	Phylum Unknown	Superkingdom Shared
20.	*Neisseria meningitidis MC58*	1000	Genus Unknown	Phylum Shared
21.	*Nostoc sp. PCC 7120*	1000	Order Unknown	Superkingdom Shared
22.	*Polaromonas sp. JS666*	1000	Species Unknown	Phylum Shared
23.	*Pseudomonas aeruginosa PAO1*	1000	Genus Unknown	Class Shared
24.	*Pseudomonas mendocina ymp*	1000	Genus Unknown	Class Shared
25.	*Rhizobium etli CFN 42*	1000	Species Unknown	Class Shared
26.	*Rhodopseudomonas palustris BisA53*	1000	Genus Unknown	Class Shared
27.	*Rickettsia bellii RML369-C*	1000	Genus Unknown	Class Shared
28.	*Saccharophagus degradans 2-40*	1000	Genus Unknown	Class Shared
29.	*Shewanella baltica OS185*	1000	Species Unknown	Class Shared
30.	*Staphylococcus aureus subsp. aureus*	1000	Species Unknown	Phylum Shared
31.	*Streptomyces coelicolor A3(2)*	1000	Species Unknown	Class Shared
32.	*Thermotoga maritima MSB8*	1000	Genus Unknown	Superkingdom Shared
33.	*Trichodesmium erythraeum IMS101*	1000	Order Unknown	Superkingdom Shared
34.	*Wigglesworthia glossinidia endosymbiont of Glossina brevipalpis*	1000	Genus Unknown	Class Shared
35.	*Xanthomonas campestris pv. campestris str. ATCC 33913*	1000	Genus Unknown	Class Shared

*All Known: Genome fragments of that particular microbe is present in the modified RefDB.
*Strain Unknown: Genome fragments of this strain are absent in the modified RefDB.
*Species Unknown: Genome fragments of all strains belonging to this species are absent in the modified RefDB.
*Genus Unknown: Genome fragments of all the strains and species belonging to this genus are absent in modified RefDB.
*Family Unknown: Genome fragments of all genera belonging to this family are absent in the modified RefDB.
*Order Unknown: Genome fragments of all families belonging to this order are absent in the modified RefDB.
*Phylum Unknown: Genome fragments of all classes belonging to this phylum are absent in modified RefDB.

**Class Shared: The training data set is devoid of genomes that can share either strain or species or genus or family or order with that particular microbe.
**Phylum Shared: The training data set does not consists of any genomes that belong to either strain or species or genus or family or order or class of that particular microbe.
**Superkingdom Shared: The training data set does not consists of any genomes that belong to either strain or species or genus or family or order or class or phylum of that particular microbe.

Supplementary Table 1: (contd)

454-100 data set				
**S. No.**	**Organism**	**Number of sequences**	**Status with respect to modified RefDB***	**Status with respect to training data set****
1.	*Acidovorax avenae*	1000	All Known	Phylum Shared
2.	*Agrobacterium tumefaciens str. C58*	1000	Species Unknown	Class Shared
3.	*Bacillus halodurans C-125*	1000	Species Unknown	Phylum Shared
4	*Bdellovibrio bacteriovorus HD100*	1000	Order Unknown	Phylum Shared
5.	*Bordetella pertussis Tohama I*	1000	Family Unknown	Phylum Shared
6.	*Bradyrhizobium sp. BTAi1*	1000	Species Unknown	Class Shared
7.	*Burkholderia cenocepacia AU 1054*	1000	All Known	Phylum Shared
8.	*Campylobacter jejuni subsp. jejuni NCTC 11168*	1000	Genus Unknown	Phylum Shared
9.	*Chlamydophila pneumoniae AR39*	1000	Genus Unknown	Superkingdom Shared
10.	*Chlorobium chlorochromatii CaD3*	1000	Genus Unknown	Superkingdom Shared
11.	*Clostridium tetani E88*	1000	Species Unknown	Class Shared
12.	*Corynebacterium diphtheriae NCTC 13129*	1000	Species Unknown	Class Shared
13.	*Francisella philomiragia subsp. philomiragia ATCC 25017*	1000	Species Unknown	Class Shared
14.	*Haemophilus influenzae Rd KW20*	1000	Species Unknown	Class Shared
15.	*Helicobacter pylori J99*	1000	Strain Unknown	Phylum Shared
16.	*Lactobacillus acidophilus NCFM*	1000	Family Unknown	Phylum Shared
17.	*Leptospira interrogans serovar Lai str. 56601*	1000	Family Unknown	Superkingdom Shared
18.	*Magnetococcus sp. MC-1*	1000	All Known	Phylum Shared
19.	*Mycoplasma pneumoniae M129*	1000	Phylum Unknown	Superkingdom Shared
20.	*Neisseria meningitidis MC58*	1000	Genus Unknown	Phylum Shared
21.	*Nostoc sp. PCC 7120*	1000	Order Unknown	Superkingdom Shared
22.	*Polaromonas sp. JS666*	1000	Species Unknown	Phylum Shared
23.	*Pseudomonas aeruginosa PAO1*	1000	Genus Unknown	Class Shared
24.	*Pseudomonas mendocina ymp*	1000	Genus Unknown	Class Shared
25.	*Rhizobium etli CFN 42*	1000	Species Unknown	Class Shared
26.	*Rhodopseudomonas palustris BisA53*	1000	Genus Unknown	Class Shared
27.	*Rickettsia bellii RML369-C*	1000	Genus Unknown	Class Shared
28.	*Saccharophagus degradans 2-40*	1000	Genus Unknown	Class Shared
29.	*Shewanella baltica OS185*	1000	Species Unknown	Class Shared
30.	*Staphylococcus aureus subsp. aureus*	1000	Species Unknown	Phylum Shared
31.	*Streptomyces coelicolor A3(2)*	1000	Species Unknown	Class Shared
32.	*Thermotoga maritima MSB8*	1000	Genus Unknown	Superkingdom Shared
33.	*Trichodesmium erythraeum IMS101*	1000	Order Unknown	Superkingdom Shared
34.	*Wigglesworthia glossinidia endosymbiont of Glossina brevipalpis*	1000	Genus Unknown	Class Shared
35.	*Xanthomonas campestris pv. campestris str. ATCC 33913*	1000	Genus Unknown	Class Shared

*All Known: Genome fragments of that particular microbe is present in the modified RefDB.
*Strain Unknown: Genome fragments of this strain are absent in the modified RefDB.
*Species Unknown: Genome fragments of all strains belonging to this species are absent in the modified RefDB.
*Genus Unknown: Genome fragments of all the strains and species belonging to this genus are absent in modified RefDB.
*Family Unknown: Genome fragments of all genera belonging to this family are absent in the modified RefDB.
*Order Unknown: Genome fragments of all families belonging to this order are absent in the modified RefDB.
*Phylum Unknown: Genome fragments of all classes belonging to this phylum are absent in modified RefDB.

**Class Shared: The training data set is devoid of genomes that can share either strain or species or genus or family or order with that particular microbe.
**Phylum Shared: The training data set does not consists of any genomes that belong to either strain or species or genus or family or order or class of that particular microbe.
**Superkingdom Shared: The training data set does not consists of any genomes that belong to either strain or species or genus or family or order or class or phylum of that particular microbe.
