# Supplementary material for: INDUS - a composition-based approach for rapid and accurate taxonomic classification of metagenomic sequences
Source: BMC Genomics. 2011 Nov 30;12(Suppl 3):S4. doi: 10.1186/1471-2164-12-S3-S4 (PMC3333187; doi:10.1186/1471-2164-12-S3-S4)
Supplement: Additional file 3 — List of organisms constituting the FAMeS data sets. A document containing the list constituting the simHC, simMC and the simLC data sets and their status with respect to the modified reference database. [file 1471-2164-12-S3-S4-S3.pdf]

Supplementary Table 2: The organisms constituting the simLC, simMC and the simHC data sets along with their representation status* with respect to modified reference database (modified RefDB) and their phylogenetic similarity status** with respect to the genomes of training data set.

S. No.	Organism	Number of sequences			Status with respect to modified RefDB*	Status with respect to training data set**
		simLC	simMC	simHC		
1	*Actinobacillus succinogenes 130Z*	252	290	483	All Known	Class Shared
2	*Alkalilimnicola ehrlichei MLHE-1*	373	384	829	All Known	Family Shared
3	*Alkaliphillus metalliredigenes UNDEF*	489	504	1091	Genus Unknown	Class Shared
4	*Anabaena variabilis ATCC 29413*	855	881	1703	Class Unknown	Superkingdom Shared
5	*Anaeromyxobacter dehalogenans 2CP-C*	584	578	1273	All Known	Phylum Shared
6	*Arthrobacter sp. FB24*	570	613	1211	All Known	Class Shared
7	*Azotobacter vinelandii AvOP*	650	652	1311	All Known	Class Shared
8	*Bacillus cereus NVH391-98*	520	454	1000	All Known	Phylum Shared
9	*Bifidobacterium longum DJO10A*	288	235	610	All Known	Species Shared
10	*Bradyrhizobium sp. BTAi1*	9277	22956	2127	All Known	Class Shared
11	*Brevibacterium linens BL2*	542	506	1088	Family Unknown	Class Shared
12	*Burkholderia ambifaria AMMD*	955	926	1937	All Known	Phylum Shared
13	*Burkholderia cenocepacia AU 1054*	879	917	1791	All Known	Phylum Shared
14	*Burkholderia cenocepacia HI2424*	956	1010	2045	All Known	Phylum Shared
15	*Burkholderia sp. sp.strain 383*	1074	1024	2191	All Known	Phylum Shared
16	*Burkholderia vietnamiensis G4*	992	985	2083	All Known	Phylum Shared
17	*Burkholderia xenovorans LB400*	1149	1146	2384	All Known	Phylum Shared
18	*Caldicellulosiruptor saccharolyticus UNDEF*	367	316	658	Order Unknown	Order Shared
19	*Chlorobium limicola DSMZ 245(T)*	381	353	671	Genus Unknown	Superkingdom Shared
20	*Chlorobium phaeobacteroides BS1*	483	515	1082	Genus Unknown	Superkingdom Shared
21	*Chlorobium phaeobacteroides DSM 266*	359	390	719	Genus Unknown	Superkingdom Shared
22	*Chlorobium vibrioforme f. thiosulfatophilum DSMZ 265(T)*	199	286	534	Genus Unknown	Superkingdom Shared
23	*Chloroflexus aurantiacus J-10-fl*	679	668	1277	All Known	Family Shared
24	*Chromohalobacter salexigens DSM3043*	454	481	888	All Known	Order Shared
25	*Clostridium beijerincki NCIMB 8052*	737	702	1411	Species Unknown	Class Shared
26	*Clostridium thermocellum ATCC 27405*	461	494	932	Species Unknown	Class Shared
27	*Crocosphaera watsonii WH 8501*	812	776	1593	Family Unknown	Superkingdom Shared
28	*Cytophaga hutchinsonii ATCC 33406*	5168	580	1161	All Known	Phylum Shared
29	*Dechloromonas aromatica RCB*	537	559	1132	All Known	Phylum Shared
30	*Deinococcus geothermalis DSM11300*	415	404	809	All Known	All Shared
31	*Desulfitobacterium hafniense DCB-2*	769	725	1486	Family Unknown	Class Shared
32	*Desulfovibrio desulfuricans G20*	484	424	919	All Known	Phylum Shared
33	*Ehrlichia canis Jake*	196	197	283	All Known	Class Shared
34	*Ehrlichia chaffeensis sapulpa*	150	155	255	All Known	Class Shared
35	*Enterococcus faecium DO*	359	360	676	Family Unknown	Phylum Shared
36	*Exiguobacterium UNDEF 255-15*	377	392	788	All Known	Phylum Shared

S. No.	Organism	Number of sequences			Status with respect to modified RefDB*	Status with respect to training data set**
		simLC	simMC	simHC		
37	*Ferroplasma acidarmanus fer1*	238	264	471	Family Unknown	Order Shared
38	*Frankia sp. CcI3*	645	643	1334	All Known	Class Shared
39	*Frankia sp. EAN1pec*	1109	1111	2248	All Known	Class Shared
40	*Geobacter metallireducens GS-15*	515	520	1025	All Known	Phylum Shared
41	*Haemophilus somnus 129PT*	232	265	513	All Known	Class Shared
42	*Jannaschia sp. CCS1*	543	543	1148	All Known	Family Shared
43	*Kineococcus radiotolerans SRS30216*	566	580	1187	All Known	Class Shared
44	*Lactobacillus brevis ATCC 367*	177	247	445	Family Unknown	Phylum Shared
45	*Lactobacillus casei ATCC 334*	362	344	648	Family Unknown	Phylum Shared
46	*Lactobacillus delbrueckii bulgaricus ATCC BAA-365*	195	203	391	Family Unknown	Phylum Shared
47	*Lactobacillus gasseri ATCC 33323*	244	258	551	Family Unknown	Phylum Shared
48	*Lactococcus lactis cremoris SK11*	301	345	584	Genus Unknown	Phylum Shared
49	*Leuconostoc mesenteroides mesenteroides ATCC 8293*	235	241	473	Family Unknown	Phylum Shared
50	*Magnetococcus sp. MC-1*	504	520	1153	All Known	Phylum Shared
51	*Marinobacter aquaeolei VT8*	547	586	1164	All Known	Class Shared
52	*Mesorhizobium sp. BNC1*	567	605	1289	All Known	Class Shared
53	*Methanococcoides burtonii DSM6242*	268	318	663	All Known	Family Shared
54	*Methanosarcina barkeri Fusaro*	545	608	1213	All Known	Genus Shared
55	*Methanospirillum hungatei JF-1*	429	459	919	All Known	Order Shared
56	*Methylobacillus flagellatus strain KT*	365	321	687	All Known	Phylum Shared
57	*Moorella thermoacetica ATCC 39073*	377	765	740	Order Unknown	Family Shared
58	*Nitrobacter hamburgensis UNDEF*	630	568	1272	All Known	Class Shared
59	*Nitrobacter winogradskyi Nb-255*	427	358	857	All Known	Class Shared
60	*Nitrosococcus oceani UNDEF*	409	406	868	All Known	Order Shared
61	*Nitrosomonas eutropha C71*	314	320	649	All Known	Phylum Shared
62	*Nitrosospira multiformis ATCC 25196*	378	420	814	All Known	Phylum Shared
63	*Nocardioides sp. JS614*	636	691	1337	All Known	Class Shared
64	*Novosphingobium aromaticivorans DSM 12444 (F199)*	520	536	1093	All Known	Family Shared
65	*Oenococcus oeni PSU-1*	182	221	422	Family Unknown	Phylum Shared
66	*Paracoccus denitrificans PD1222*	585	620	1362	All Known	Family Shared
67	*Pediococcus pentosaceus ATCC 25745*	217	173	456	Family Unknown	Phylum Shared
68	*Pelobacter carbinolicus DSM 2380*	489	472	896	All Known	Phylum Shared
69	*Pelobacter propionicus DSM 2379*	508	550	1145	All Known	Phylum Shared
70	*Pelodictyon luteolum UNDEF*	250	314	581	Genus Unknown	Superkingdom Shared
71	*Pelodictyon phaeoclathratiforme BU-1 (DSMZ 5477(T))*	402	359	703	Genus Unknown	Superkingdom Shared
72	*Polaromonas sp. JS666*	733	769	1489	Species Unknown	Phylum Shared
73	*Prochlorococcus marinus str. MIT 9312*	183	218	404	All Known	Superkingdom Shared
74	*Prochlorococcus sp. NATL2A*	253	191	480	All Known	Superkingdom Shared
75	*Prosthecochloris aestuarii SK413/DSMZ 271(t)*	282	269	692	Species Unknown	Superkingdom Shared
76	*Pseudoalteromonas atlantica T6c*	588	657	1301	All Known	Class Shared

S. No.	Organism	Number of sequences			Status with respect to modified RefDB*	Status with respect to training data set**
		simLC	simMC	simHC		
77	*Pseudomonas fluorescens PfO-1*	730	791	1587	Genus Unknown	Class Shared
78	*Pseudomonas putida F1*	675	745	1528	Genus Unknown	Class Shared
79	*Pseudomonas syringae B728a*	746	738	1545	Genus Unknown	Class Shared
80	*Psychrobacter arcticum 273-4*	327	329	623	All Known	Class Shared
81	*Psychrobacter cryopegella UNDEF*	422	366	793	All Known	Class Shared
82	*Rhodobacter sphaeroides 2.4.1*	514	528	1119	All Known	Species Shared
83	*Rhodoferax ferrireducens UNDEF*	599	588	1276	All Known	Phylum Shared
84	*Rhodopseudomonas palustris BisA53*	636	680	1392	Genus Unknown	Class Shared
85	*Rhodopseudomonas palustris BisB18*	699	6107	1348	Genus Unknown	Class Shared
86	*Rhodopseudomonas palustris BisB5*	575	16577	1200	Genus Unknown	Class Shared
87	*Rhodopseudomonas palustris HaA2*	28861	671	1339	Genus Unknown	Class Shared
88	*Rhodospirillum rubrum ATCC 11170*	559	4868	1062	All Known	All Shared
89	*Rubrobacter xylanophilus DSM 9941*	409	444	799	All Known	All Shared
90	*Ruegeria sp. TM1040*	469	470	1065	Species Unknown	Family Shared
91	*Saccharophagus degradans 2-40*	582	642	1324	Genus Unknown	Class Shared
92	*Shewanella amazonensis SB2B*	536	482	1055	All Known	Class Shared
93	*Shewanella baltica OS155*	621	649	1313	Species Unknown	Class Shared
94	*Shewanella frigidimarina NCMB400*	551	559	1257	All Known	Class Shared
95	*Shewanella putefaciens UNDEF*	565	530	1153	All Known	Class Shared
96	*Shewanella sp. ANA-3*	664	586	1279	All Known	Class Shared
97	*Shewanella sp. MR-7*	568	515	1177	All Known	Class Shared
98	*Shewanella sp. PV-4*	524	537	1165	All Known	Class Shared
99	*Shewanella sp. W3-18-1*	533	532	1214	All Known	Class Shared
100	*Sphingopyxis alaskensis RB2256*	438	402	846	All Known	Family Shared
101	*Streptococcus suis 89/1591*	263	276	490	Species Unknown	Phylum Shared
102	*Streptococcus thermophilus LMD-9*	178	187	501	Species Unknown	Phylum Shared
103	*Sulfurimonas denitrificans DSM 1251*	277	312	490	All Known	Phylum Shared
104	*Synechococcus sp. PCC 7942 (elongatus)*	316	309	646	All Known	Superkingdom Shared
105	*Syntrophobacter fumaroxidans MPOB*	606	552	1181	All Known	Phylum Shared
106	*Syntrophomonas wolfei Goettingen*	314	363	708	Family Unknown	Class Shared
107	*Thermoanaerobacter ethanolicus 39E*	315	265	570	Order Unknown	All Shared
108	*Thermobifida fusca YX*	434	431	930	All Known	Class Shared
109	*Thiobacillus denitrificans ATCC 25259*	395	400	741	All Known	Phylum Shared
110	*Thiomicrospira crunogena XCL-2*	274	296	603	Family Unknown	Class Shared
111	*Trichodesmium erythraeum IMS101*	977	927	2051	Class Unknown	Superkingdom Shared
112	*Xylella fastidiosa Dixon*	601	10484	1303	All Known	Class Shared
	Total Number of Reads	97495	114834	116771		
	Total Number of Reads Considered after exclusion of low length reads***	96732	113373	115592		

*All Known: Genome fragments of that particular microbe is present in the modified RefDB.
*Strain Unknown: Genome fragments of this strain are absent in the modified RefDB.
*Species Unknown: Genome fragments of all strains belonging to this species are absent in the modified RefDB.
*Genus Unknown: Genome fragments of all the strains and species belonging to this genus are absent in modified RefDB.
*Family Unknown: Genome fragments of all genera belonging to this family are absent in the modified RefDB.
*Order Unknown: Genome fragments of all families belonging to this order are absent in the modified RefDB.
*Phylum Unknown: Genome fragments of all classes belonging to this phylum are absent in modified RefDB.

**All Shared: The training data set includes the sequences from the genome of that particular microbe.
**Species Shared: The training data set is devoid of genomes belonging to the strain of that particular microbe
**Genus Shared: The training data set is devoid of genomes that can share either strain or species with that particular microbe.
**Family Shared: The training data set does not consists of any genomes that belong to either strain or species or genus of that particular microbe.
**Order Shared: The training data set does not consists of any genomes that belong to either strain or species or genus or family of that particular microbe.
**Class Shared: The training data set is devoid of genomes that can share either strain or species or genus or family or order with that particular microbe.
**Phylum Shared: The training data set does not consists of any genomes that belong to either strain or species or genus or family or order or class of that particular microbe.
**Super-kingdom Shared: The training data set does not consists of any genomes that belong to either strain or species or genus or family or order or class or phylum of that particular microbe.

***Sequences having a length less than 400bp were removed from all three data sets.
