# Supplementary material for: INDUS - a composition-based approach for rapid and accurate taxonomic classification of metagenomic sequences
Source: BMC Genomics. 2011 Nov 30;12(Suppl 3):S4. doi: 10.1186/1471-2164-12-S3-S4 (PMC3333187; doi:10.1186/1471-2164-12-S3-S4)
Supplement: Additional File 4 — Detailed results of validation on the simulated test data sets A document summarizing the pattern of taxonomic assignments and the time taken by INDUS, TACOA, SOrt-ITEMS, MEGAN and SPHINX on the four simulated test data sets. [file 1471-2164-12-S3-S4-S4.pdf]

**Section A:** Average time (minutes) taken by various methods for binning validation data sets (constituted of 35,000 sequences each). All values were estimated using a desktop (having an Intel Xeon quad core processor and 4Gb RAM).

| Validation data set | Binning Time (in minutes) | | | | |
|---|---|---|---|---|---|
| | INDUS | TACOA | SOrt-ITEMS | MEGAN | SPHINX |
| **Sanger** | 49 | 603 | 1486 | 1336 | 74 |
| **454-400** | 45 | 514 | 1264 | 1161 | 69 |
| **454-250** | 39 | 326 | 1116 | 998 | 54 |
| **454-100** | 31 | 176 | 1044 | 971 | 42 |

**Section B:** Summarized binning results obtained for all four validation data sets with INDUS, TACOA, SOrt-ITEMS, MEGAN and SPHINX. All numbers given in this table are with respect to the total number of sequences in the respective data set.

**SANGER data set**

| Assignment category | Percentage of sequences assigned | | | | |
|---|---|---|---|---|---|
| | INDUS | TACOA | SOrt-ITEMS | MEGAN | SPHINX |
| Total correct | 71.14 | 64.82 | 80.31 | 52.06 | 70.2 |
| Wrong | 9.64 | 22.13 | 3.69 | 35.39 | 5.31 |
| Specific# levels | 49.81 | 7.92 | 64.28 | 33.73 | 55.76 |
| Non Specific* levels | 21.33 | 56.9 | 16.04 | 18.33 | 14.44 |
| Unassigned | 19.22 | 13.05 | 15.99 | 12.55 | 24.49 |

| Assignment category | Number of sequences assigned | | | | |
|---|---|---|---|---|---|
| | INDUS | TACOA | SOrt-ITEMS | MEGAN | SPHINX |
| Total correct | 24899 | 22687 | 28108.5 | 18221 | 24570 |
| Wrong | 3374 | 7745.5 | 1291.5 | 12386.5 | 1858.5 |
| Specific# levels | 17433.5 | 2772 | 22498 | 11805.5 | 19516 |
| Non Specific* levels | 7465.5 | 19915 | 5614 | 6415.5 | 5054 |
| Unassigned | 6727 | 4567.5 | 5596.5 | 4392.5 | 8571.5 |

| Assignment category | Time taken (in minutes) | | | | |
|---|---|---|---|---|---|
| | INDUS | TACOA | SOrt-ITEMS | MEGAN | SPHINX |
| Total correct | 35 | 391 | 1193 | 696 | 52 |
| Wrong | 5 | 133 | 55 | 473 | 4 |
| Specific# levels | 24 | 48 | 955 | 451 | 41 |
| Non Specific* levels | 10 | 343 | 238 | 245 | 11 |
| Unassigned | 9 | 79 | 238 | 168 | 18 |

**454-400 data set**

| Assignment category | Percentage of sequences assigned | | | | |
|---|---|---|---|---|---|
| | INDUS | TACOA | SOrt-ITEMS | MEGAN | SPHINX |
| Total correct | 61.15 | 58.75 | 71.39 | 47.41 | 60.39 |
| Wrong | 10.04 | 24.99 | 9.93 | 36.24 | 12.03 |
| Specific# levels | 37.33 | 8.41 | 56.63 | 29.9 | 46.7 |
| Non Specific* levels | 23.82 | 50.33 | 14.77 | 17.51 | 13.69 |
| Unassigned | 28.81 | 16.27 | 18.67 | 16.35 | 27.58 |

| Assignment category | Number of sequences assigned | | | | |
|---|---|---|---|---|---|
| | INDUS | TACOA | SOrt-ITEMS | MEGAN | SPHINX |
| Total correct | 21402.5 | 20562.5 | 24986.5 | 16593.5 | 21136.5 |
| Wrong | 3514 | 8746.5 | 3475.5 | 12684 | 4210.5 |
| Specific# levels | 13065.5 | 2943.5 | 19820.5 | 10465 | 16345 |
| Non Specific* levels | 8337 | 17615.5 | 5169.5 | 6128.5 | 4791.5 |
| Unassigned | 10083.5 | 5694.5 | 6534.5 | 5722.5 | 9653 |

| Assignment category | Time taken (in minutes) | | | | |
|---|---|---|---|---|---|
| | INDUS | TACOA | SOrt-ITEMS | MEGAN | SPHINX |
| Total correct | 28 | 302 | 902 | 550 | 42 |
| Wrong | 5 | 128 | 126 | 421 | 8 |
| Specific# levels | 17 | 43 | 716 | 347 | 32 |
| Non Specific* levels | 11 | 259 | 187 | 203 | 9 |
| Unassigned | 13 | 84 | 236 | 190 | 19 |

(contd ..)

**454-250 data set**

| Assignment category | Percentage of sequences assigned | | | | |
|---|---|---|---|---|---|
| | **INDUS** | **TACOA** | **SOrt-ITEMS** | **MEGAN** | **SPHINX** |
| Total correct | 54.79 | 53.63 | 56.99 | 40.95 | 47.49 |
| Wrong | 10.86 | 27.99 | 7.21 | 25.93 | 7 |
| Specific# levels | 32.74 | 6.81 | 43.23 | 26.88 | 36 |
| Non Specific* levels | 22.05 | 46.82 | 13.76 | 14.07 | 11.49 |
| Unassigned | 34.36 | 18.39 | 35.79 | 33.12 | 45.51 |

| Assignment category | Number of sequences assigned | | | | |
|---|---|---|---|---|---|
| | **INDUS** | **TACOA** | **SOrt-ITEMS** | **MEGAN** | **SPHINX** |
| Total correct | 19176.5 | 18770.5 | 19946.5 | 14332.5 | 16621.5 |
| Wrong | 3801 | 9796.5 | 2523.5 | 9075.5 | 2450 |
| Specific# levels | 11459 | 2383.5 | 15130.5 | 9408 | 12600 |
| Non Specific* levels | 7717.5 | 16387 | 4816 | 4924.5 | 4021.5 |
| Unassigned | 12026 | 6436.5 | 12526.5 | 11592 | 15928.5 |

| Assignment category | Time taken (in minutes) | | | | |
|---|---|---|---|---|---|
| | **INDUS** | **TACOA** | **SOrt-ITEMS** | **MEGAN** | **SPHINX** |
| Total correct | 21 | 175 | 636 | 409 | 26 |
| Wrong | 4 | 91 | 80 | 259 | 4 |
| Specific# levels | 13 | 22 | 482 | 268 | 19 |
| Non Specific* levels | 9 | 153 | 154 | 140 | 6 |
| Unassigned | 13 | 60 | 399 | 331 | 25 |

(contd ..)

**454-100 data set**

| Assignment category | Percentage of sequences assigned | | | | |
|---|---|---|---|---|---|
| | INDUS | TACOA | SOrt-ITEMS | MEGAN | SPHINX |
| Total correct | 41.72 | 45.05 | 35.87 | 19.99 | 37.94 |
| Wrong | 8.58 | 31.66 | 5.43 | 23.05 | 7.46 |
| Specific# levels | 23.73 | 4.33 | 31.07 | 15.43 | 24.43 |
| Non Specific* levels | 17.99 | 40.72 | 4.8 | 4.56 | 13.51 |
| Unassigned | 49.7 | 23.29 | 58.7 | 56.96 | 54.61 |

| Assignment category | Number of sequences assigned | | | | |
|---|---|---|---|---|---|
| | INDUS | TACOA | SOrt-ITEMS | MEGAN | SPHINX |
| Total correct | 14602 | 15767.5 | 12554.5 | 6996.5 | 13279 |
| Wrong | 3003 | 11081 | 1900.5 | 8067.5 | 2611 |
| Specific# levels | 8305.5 | 1515.5 | 10874.5 | 5400.5 | 8550.5 |
| Non Specific* levels | 6296.5 | 14252 | 1680 | 1596 | 4728.5 |
| Unassigned | 17395 | 8151.5 | 20545 | 19936 | 19113.5 |

| Assignment category | Time taken (in minutes) | | | | |
|---|---|---|---|---|---|
| | INDUS | TACOA | SOrt-ITEMS | MEGAN | SPHINX |
| Total correct | 13 | 79 | 374 | 194 | 16 |
| Wrong | 3 | 56 | 57 | 224 | 3 |
| Specific# levels | 7 | 8 | 324 | 150 | 10 |
| Non Specific* levels | 6 | 72 | 50 | 44 | 6 |
| Unassigned | 15 | 41 | 613 | 553 | 23 |
