# Supplementary material for: INDUS - a composition-based approach for rapid and accurate taxonomic classification of metagenomic sequences
Source: BMC Genomics. 2011 Nov 30;12(Suppl 3):S4. doi: 10.1186/1471-2164-12-S3-S4 (PMC3333187; doi:10.1186/1471-2164-12-S3-S4)
Supplement: Additional file 5 — Time performance of the INDUS algorithm A document containing the time taken by INDUS for binning 10000, 20000, 100000 and 500000 sequences. [file 1471-2164-12-S3-S4-S5.pdf]

Average time (minutes) taken by INDUS for binning 10000, 20000, 50000, 100000, 500000 sequences. All values were estimated using a desktop with the following specifications - Intel Xeon quad core processor and 4 GB RAM.

Test data set 1: Average length of sequences: 800 bp

Total number of sequences in test data set	Time taken for binning (minutes)
**10000**	18
**20000**	29
**50000**	62
**100000**	136
**500000**	753

Test data set 2: Average length of sequences: 400 bp

Total number of sequences in test data set	Time taken for binning (minutes)
**10000**	16
**20000**	28
**50000**	60
**100000**	127
**500000**	741

Test data set 3: Average length of sequences: 250 bp

Total number of sequences in test data set	Time taken for binning (minutes)
**10000**	11
**20000**	20
**50000**	53
**100000**	119
**500000**	668

Test data set 4: Average length of sequences: 100 bp

Total number of sequences in test data set	Time taken for binning (minutes)
**10000**	9
**20000**	17
**50000**	48
**100000**	99
**500000**	641
