# Supplementary material for: INDUS - a composition-based approach for rapid and accurate taxonomic classification of metagenomic sequences
Source: BMC Genomics. 2011 Nov 30;12(Suppl 3):S4. doi: 10.1186/1471-2164-12-S3-S4 (PMC3333187; doi:10.1186/1471-2164-12-S3-S4)
Supplement: Additional File 6 — Detailed results of validation on the FAMeS data sets A document containing the summarized results of (A) INDUS (B) TACOA (C)SOrt-ITEMS (D) MEGAN and (E) SPHINX obtained for the simLC, simMC and simHC data sets. [file 1471-2164-12-S3-S4-S6.pdf]

Summarized results of (A) INDUS (B) TACOA (C) SOrt-ITEMS (D) MEGAN and (E) SPHINX, obtained for simLC, simMC, and simHC data sets. All numbers given in this table are with respect to the total number of sequences in the respective data set.

(A) INDUS

Assignment Category/Taxon	Percentage of Sequences Assigned					
	SimLC		SimMC		SimHC	
	Complete RefDB[@]	Modified RefDB[$]	Complete RefDB[@]	Modified RefDB[$]	Complete RefDB[@]	Modified RefDB[$]
**Non-Specific* Levels**	8.16	14.75	8.5	14.03	13.06	15.25
**Specific[#] Levels**	81.38	66.75	84.26	72.12	76.58	63.65
**Phylum**	15.61	21.51	14.85	19.82	13.06	13.53
**Class**	4.73	12.77	7.2	14.55	6.21	9.1
**Order**	3.24	7.08	3.81	7.56	2.9	3.39
**Family**	8.87	6.27	10.42	7.97	10.69	8.03
**Genus & Lower Levels**	48.93	19.12	47.98	22.21	43.72	29.6
**Total Correct**	89.54	81.49	92.76	86.15	89.64	78.91
**Wrong**	2.61	7.28	1	4.51	1.89	5.8
**Unassigned**	7.85	11.23	6.24	9.34	8.47	15.29

(B) TACOA

Assignment Category/Taxon	Percentage of Sequences Assigned					
	SimLC		SimMC		SimHC	
	Complete RefDB[@]	Modified RefDB[$]	Complete RefDB[@]	Modified RefDB[$]	Complete RefDB[@]	Modified RefDB[$]
**Non-Specific* Levels**	54.9	53.5	58.8	56.0	53.4	48.8
**Specific[#] Levels**	24.4	21.0	23.7	23.0	18.7	18.2
**Phylum**	19.7	15.3	19.0	16.2	11.3	8.9
**Class**	0.9	1.6	1.3	2.6	1.6	2.6
**Order**	0.8	0.9	0.8	1.1	0.9	1.2
**Family**	0.04	1.04	0.02	1.0	0.1	1.1
**Genus & Lower Levels**	2.89	2.13	2.6	2.1	4.8	4.5
**Total Correct**	79.3	74.4	82.5	79.0	72.1	67.0
**Wrong**	9.0	13.9	7.3	10.8	11.4	16.0
**Unassigned**	11.6	11.6	10.2	10.2	16.5	17.0

*Non-specific: Assignments made at super kingdom level
[#]Specific : Assignments made at phylum, class, order, family and genus levels
[@]Complete RefDB: Complete reference database constructed from sequences belonging to 952 genomes
[$]Modified RefDB: Modified reference database created by removing sequences of 300 genomes from the complete reference database
Note that the subtotals may vary by a value of 0.1, since the individual values are rounded off to single decimals.

(C) SOrt-ITEMS

Assignment Category/Taxon	Percentage of Sequences Assigned					
	SimLC		SimMC		SimHC	
	Complete RefDB@	Modified RefDB$	Complete RefDB@	Modified RefDB$	Complete RefDB@	Modified RefDB$
Non-Specific* Levels	0.0	0.0	0.0	0.0	0.0	0.0
Specific# Levels	94.9	78.1	93.7	79.1	92.0	78.1
Phylum	2.4	10.5	2.3	8.8	3.7	9.1
Class	2.6	12.4	3.0	10.9	4.0	8.4
Order	0.2	0.1	0.2	0.1	0.3	0.1
Family	5.0	17.1	5.3	16.5	6.4	7.8
Genus & Lower Levels	84.6	37.9	83.0	42.9	77.6	52.7
Total Correct	94.9	78.1	93.7	79.1	92.0	78.1
Wrong	2.0	9.2	3.1	9.1	3.6	9.2
Unassigned	3.1	12.6	3.2	11.8	4.3	12.6

(D) MEGAN

Assignment Category/Taxon	Percentage of Sequences Assigned					
	SimLC		SimMC		SimHC	
	Complete RefDB@	Modified RefDB$	Complete RefDB@	Modified RefDB$	Complete RefDB@	Modified RefDB$
Non-Specific* Levels	1.1	7.6	1.0	6.7	1.5	8.4
Specific# Levels	93.9	65.0	93.1	63.2	90.3	69.5
Phylum	0.8	5.3	0.7	4.6	3.7	3.9
Class	0.6	2.7	0.6	2.7	4.0	2.2
Order	1.4	4.1	1.2	3.3	0.3	1.6
Family	5.4	13.4	5.4	11.1	4.4	2.9
Genus & Lower Levels	85.8	39.6	85.1	41.5	77.9	58.9
Total Correct	95.0	72.6	94.0	69.9	91.9	75.9
Wrong	2.7	23.6	3.5	26.6	4.9	17.6
Unassigned	2.3	3.7	2.4	3.5	3.2	4.5

*Non-specific: Assignments made at super kingdom level
#Specific : Assignments made at phylum, class, order, family and genus levels
@Complete RefDB: Complete reference database constructed from sequences belonging to 952 genomes
$Modified RefDB: Modified reference database created by removing sequences of 300 genomes from the complete reference database
Note that the subtotals may vary by a value of 0.1, since the individual values are rounded off to single decimals.

(E) SPHINX

Assignment Category/Taxon	Percentage of Sequences Assigned					
	SimLC		SimMC		SimHC	
	Complete RefDB[@]	Modified RefDB[$]	Complete RefDB[@]	Modified RefDB[$]	Complete RefDB[@]	Modified RefDB[$]
**Non-Specific* Levels**	10.3	12.7	11.5	11.6	6.4	6.4
**Specific[#] Levels**	82.8	71.0	81.1	70.1	79.6	71.2
**Phylum**	4.1	13.7	5.9	17.4	5.1	12.0
**Class**	5.4	14.6	4.4	16.2	6.1	13.9
**Order**	0.8	0.2	0.5	0.1	0	0.5
**Family**	14.6	17.4	16.1	12.5	18.2	17.2
**Genus & Lower Levels**	57.9	25.1	54.2	23.9	50.2	27.6
**Total Correct**	93.1	83.7	92.6	81.8	86.0	77.6
**Wrong**	2.8	11.5	3.6	13.5	7.1	12.1
**Unassigned**	4.1	4.8	3.8	4.7	6.9	10.3

*Non-specific: Assignments made at super kingdom level
[#]Specific : Assignments made at phylum, class, order, family and genus levels
[@]Complete RefDB: Complete reference database constructed from sequences belonging to 952 genomes
[$]Modified RefDB: Modified reference database created by removing sequences of 300 genomes from the complete reference database
Note that the subtotals may vary by a value of 0.1, since the individual values are rounded off to single decimals.
