# Supplementary material for: INDUS - a composition-based approach for rapid and accurate taxonomic classification of metagenomic sequences
Source: BMC Genomics. 2011 Nov 30;12(Suppl 3):S4. doi: 10.1186/1471-2164-12-S3-S4 (PMC3333187; doi:10.1186/1471-2164-12-S3-S4)
Supplement: Additional File 7 — Probable taxonomic assignment patterns for Horizontal Gene Transfer (HGT) regions A document summarizing the probable pattern of taxonomic assignment (obtained using INDUS and a 'one-genome-one-composition' model based method) for sequences that originate from genomic regions involved in lateral gene transfer events. [file 1471-2164-12-S3-S4-S7.pdf]

Probable pattern of taxonomic assignment of sequences that originate from genomic regions involved in lateral gene transfer events. The table attempts to summarize the taxonomic assignment patterns of such sequences based on their origin, as well as, the presence/absence of fragments corresponding to the donor/recipient genomes in the reference database.

**Source genome from where the query sequence has originated**	**Presence (P) or Absence (A) of donor or recipient genomes fragments/models in reference database**		**Probable assignment pattern using**	
	**Donor genome**	**Recipient genome**	**INDUS**	**'One-genome one-composition' model based approaches**
**Donor genome**	P	P	Higher level taxon (Correct but non-specific)	Donor genome (Correct and specific)
	P	A	Donor (Correct and specific)	Donor (Correct and specific)
	A	P	Recipient (Incorrect)	Unassigned
**Recipient genome**	P	P	Higher level taxon (Correct but non-specific)	Donor (Incorrect)
	A	P	Recipient (Correct and specific)	Unassigned
	P	A	Donor (Incorrect)	Donor (Incorrect)
