# Supplementary material for: INDUS - a composition-based approach for rapid and accurate taxonomic classification of metagenomic sequences
Source: BMC Genomics. 2011 Nov 30;12(Suppl 3):S4. doi: 10.1186/1471-2164-12-S3-S4 (PMC3333187; doi:10.1186/1471-2164-12-S3-S4)
Supplement: Additional File 8 — Similarities/dissimilarities between INDUS and SPHINX A document summarizing the similarities/dissimilarities in the overall taxonomic assignment procedure adopted by INDUS and SPHINX. [file 1471-2164-12-S3-S4-S8.pdf]

A summary of the similarities/differences in the overall taxonomic assignment procedure adopted by INDUS and SPHINX

Steps of taxonomic assignment	INDUS	SPHINX
**Search space reduction**	Achieved by using a pre-clustered reference database.	Achieved by using a pre-clustered reference database.
**Identification of a subset of most similar fragments in reference database**	Uses compositional distance as a metric	Uses sequence similarity (in terms of alignment characteristics)
**Identification of an appropriate taxonomic level of assignment**	Uses compositional distance as a metric	Uses alignment parameters as a metric
**Final assignment**	Based on taxonomic convergence of obtained hits	Based on sequence orthology between query and hit sequences
